# *In vitro* model of human subcutaneous adipocyte spheroids for studying mitochondrial dysfunction and mitochondria activating compounds

**DOI:** 10.1016/j.isci.2025.114480

**Published:** 2025-12-29

**Authors:** Anita Wagner, Juulia H. Lautaoja-Kivipelto, Kalle Pehkonen, Antti Hassinen, Minna Kuusela, Lisa Röttger, Elena Herbers, Anna Ioannidou, Sophia Mädler, Ina Rothenaigner, Soumya Srinivasan, Sini Laasonen, M. Tanvir Rahman, Pinja Elomaa, Saija Kortetjärvi, Anneli Olsson, Olavi Ukkola, Kamyar Hadian, Matthias Mann, Hilkka Peltoniemi, Kirsi H. Pietiläinen, Martin Klingenspor, Kirsi A. Virtanen, Carolina E. Hagberg, Eija Pirinen

**Affiliations:** 1 Research Program for Clinical and Molecular Metabolism, Faculty of Medicine, University of Helsinki, 00014 Helsinki, Finland; 2 Research Unit of Biomedicine and Internal Medicine, Faculty of Medicine University of Oulu, 90014 Oulu, Finland; 3 Turku PET Centre, Turku University Hospital, 20520 Turku, Finland; 4 Chair for Molecular Nutritional Medicine, Technical University of Munich, TUM School of Life Sciences Weihenstephan, 85354 Freising, Germany; 5 EKFZ - Else Kröner-Fresenius-Center for Nutritional Medicine, Technical University of Munich, 80992 Munich, Germany; 6 ZIEL - Institute for Food & Health, Technical University of Munich, 85354 Freising, Germany; 7 Institute for Molecular Medicine Finland, FIMM, University of Helsinki, 00014 Helsinki, Finland; 8 Division of Cardiovascular Medicine, Department of Medicine Solna, Karolinska Institutet, 17177 Stockholm, Sweden; 9 Center for Molecular Medicine, Karolinska University Hospital, 17176 Stockholm, Sweden; 10 Department of Proteomics and Signal Transduction, Max Planck Institute of Biochemistry, 82152 Martinsried, Germany; 11 Research Unit Signaling and Translation, Helmholtz Zentrum München, 85764 Neuherberg, Germany; 12 Folkhälsan Research Center, 00290 Helsinki, Finland; 13 Eira Hospital, 00150 Helsinki, Finland; 14 Obesity Research Unit, Faculty of Medicine, University of Helsinki, 00014 Helsinki, Finland; 15 Abdominal Center, Healthy Weight Hub, Helsinki University Hospital, 00290 Helsinki, Finland; 16 Turku PET Centre, University of Turku, 20520 Turku, Finland; 17 Medical Research Center Oulu, Oulu University Hospital and University of Oulu, 90014 Oulu, Finland; 18 Biocenter Oulu, University of Oulu, 90014 Oulu, Finland

**Keywords:** Lipid, Human metabolism, Adipocyte, Mitochondria, Spheroid, Obesity

## Abstract

Mitochondrial abnormalities drive subcutaneous white adipose tissue dysfunction in obesity, yet *in vitro* models to study adipocyte mitochondria remain limited. Here, we establish a human subcutaneous adipocyte spheroid model to characterize mitochondrial metabolism under obesity-relevant conditions and drug exposure. Human preadipocyte spheroids were differentiated in ultra-low attachment plates for 3 weeks using thiazolidinedione-free medium. Matrigel embedding was incorporated into the protocol as it promoted mitochondrial network and respiration compared to scaffold-free conditions. Differentiated spheroids showed increased lipid accumulation, adipogenic gene expression, mitochondrial respiration, adiponectin secretion, and hormonal responsiveness. Lipid mixture administration during differentiation induced metabolic disturbances, including mitochondrial respiration failure, alongside increased mitochondrial biogenesis. Post-differentiation treatment with rosiglitazone, a peroxisome proliferator-activated receptor γ agonist, improved mitochondrial bioenergetics and adiponectin secretion in lipid mixture-administered adipocyte spheroids. Our model enables precise measurement of adipocyte mitochondria metabolism, providing a platform for mitochondria-focused research and drug discovery in obesity.

## Highlights

• Human adipocyte spheroids mimic mitochondrial metabolism in diverse states
• Lipid overload induces mitochondrial bioenergetic defects in adipocyte spheroids
• Early effects of PPARγ agonist include restored adipocyte mitochondrial respiration
• This model supports research on mitochondrial biology and drug targeting

## Graphical abstract

**Figure:**
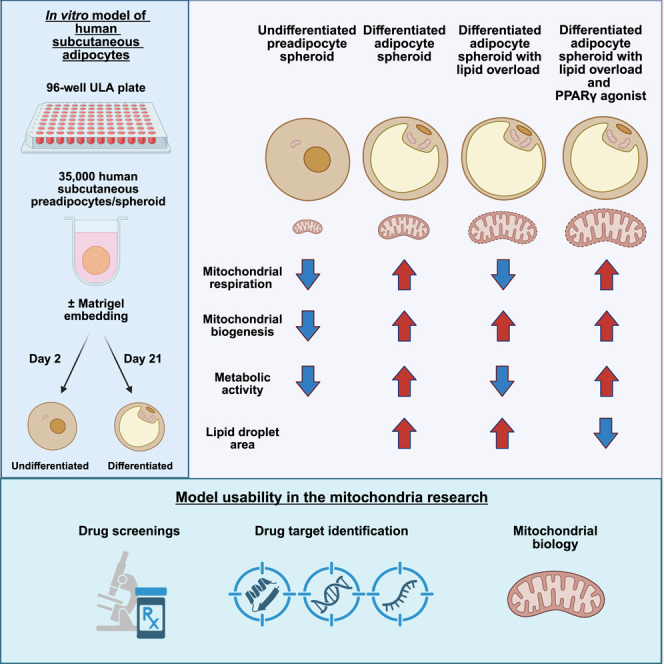

## Introduction

Subcutaneous white adipose tissue (scWAT) represents the major triglyceride store of the body. The metabolic and endocrine functions of scWAT play a crucial role in regulating energy balance and controlling systemic lipid and glucose homeostasis.^1^ Fatty acids are mobilized from lipid droplets by lipolysis to fuel the energy demand in other tissues, while adipokines secreted by adipocytes improve fatty acid and glucose utilization in target tissues.^1^ In obesity, scWAT functions are disturbed when adipocyte triglyceride storage exceeds expansion capacity, leading to ectopic lipid accumulation and subsequently to the development of metabolic complications, such as insulin resistance in non-adipose tissues.^2^ Thus, treatments restoring the key metabolic and endocrine functions in scWAT could help to reconstitute metabolic health in obesity.

One of the key factors triggering scWAT dysfunction in obesity is mitochondrial abnormalities.^3^ Mitochondrial alterations, including reduced mitochondrial respiration, number, and mass, as well as disrupted dynamics, have been reported in scWAT of humans and rodents.^3^^,^^4^ ScWAT mitochondrial dysfunction has been proposed to be a consequence of chronic energy surplus-induced inflammation.^3^ However, the understanding of the pathological causes leading to the development of scWAT mitochondrial dysfunction has remained incomplete. As scWAT mitochondrial dysfunction occurs in the early-stage of weight gain and obesity,^5^ even before the onset of obesity-related metabolic complications,^6^ it is plausible that the malfunction of scWAT mitochondria contributes to the deterioration of the whole-body glucose and lipid homeostasis. In support, the activation of tissue mitochondrial metabolism protects against obesity-related metabolic complications in mice.^7^ Clinically, pharmacological targeting of mitochondria is still an unmet need with great potential health benefits. Overall, the identification of the molecular mechanisms resulting in scWAT mitochondrial dysfunction, as well as mitochondria activating compounds, could markedly advance the drug development for obesity-related metabolic complications.

One of the main limitations in obesity research and the drug discovery field has been the lack of physiological *in vitro* models for human white subcutaneous adipocytes. The current *in vitro* research on human subcutaneous adipocytes mostly relies on the differentiation of precursor cells and preadipocytes into lipid-laden adipocytes using two-dimensional (2D) monolayer cultures.^8^ However, human precursor cells and preadipocytes fail efficiently to differentiate and mature into white adipocytes with an unilocular lipid droplet morphology and metabolic function in these monolayer cultures. For example, differentiated adipocytes can acquire brown adipocyte-like properties, such as multilocular lipid droplet morphology, high thermogenic activity, and/or uncoupling protein 1 (UCP1) expression.^9^^,^^10^ In monolayer cultures, these brown adipocyte-like properties can be caused by the use of high concentrations of thiazolidinediones, such as peroxisome proliferator-activated receptor γ (PPARγ) agonist rosiglitazone, which promotes adipocyte differentiation. By solving some of these technical challenges, three-dimensional (3D) culture models hold great promise over traditional 2D monolayer cultures.^9^^,^^11^ Several recently developed human white adipocyte spheroid models^9^^,^^12^ report 3D cultured adipocytes to better resemble their *in vivo* counterparts, showing more unilocular lipid droplet morphology and higher expression of adipogenic genes and adipokine secretion in comparison to 2D monolayer cultures.^11^ However, none of the previously developed 3D models of human white adipocytes have been specifically optimized to study mitochondrial (dys)function and testing of mitochondria-targeting pharmacological compounds.

The objective of this study was to establish a human subcutaneous white adipocyte spheroid culture model that allows the investigation of mitochondrial variables under various metabolic conditions and in response to mitochondrial-activating drugs. We demonstrate that our model effectively mimics mitochondrial remodeling associated with adipogenic differentiation and lipid overloading-induced mitochondrial dysfunction. This enables the assessment of human white subcutaneous adipocyte mitochondrial biology in both physiological and pathophysiological contexts. Moreover, we present our adipocyte spheroid model as a valuable *in vitro* platform for the investigation of pharmacological compounds that may influence mitochondrial function, thereby advancing novel drug discovery and deepening our understanding of disease mechanisms.

## Results

### Optimization of the cell culture conditions and differentiation protocol

To create a human adipocyte 3D model for studying mitochondrial function, we started by optimizing differentiation conditions using preadipocytes from two donors with different body mass index (BMI) (donors 1 and 2, Table 1). The preadipocytes were first expanded in monolayer cultures in growth medium supplemented with human serum to avoid exposure to xenobiotic compounds and then transferred to 3D culture plates. Differentiation was induced with commercially available chemically defined differentiation and maturation medium devoid of serum. This approach was chosen as human adipose-derived precursor cells and preadipocytes do not require serum for differentiation.^13^^,^^14^^,^^15^ Moreover, PPARγ agonist thiazolidinedione-free differentiation medium was used throughout all experiments to preserve the white adipocyte phenotype upon differentiation.

**Table 1:** The donor characteristics for subcutaneous preadipocytes, MAFs, and scWAT biopsies

	Cell/tissue type donated	Sex	Age (years)	Weight (kg)	Height (cm)	BMI
Donor 1	Preadipocytes	Female	43	65	168	23
Donor 2	Preadipocytes	Female	42	77	165	28
Donor 3	scWAT/MAF	Female	43	66	161	26
Donor 4	MAF	Female	52	68	165	25
Donor 5	MAF	Female	49	90	174	30
Donor 6	scWAT	Female	40	81	170	28
Donor 7	scWAT/MAF	Female	36	53	168	19
Donor 8	scWAT/MAF	Female	53	57	156	23
Donor 9	scWAT/MAF	Female	51	59	156	24

Age is reported at the time of the sample collection.

To find the optimal adipocyte spheroid size and differentiation duration, we seeded 15,000, 25,000, or 35,000 preadipocytes per well and differentiated the spheroids under scaffold-free conditions for 6, 13, or 20 days in differentiation medium, followed by a 7-day culture in maintenance medium (Figure S1A). This resulted in a 2-, 3-, or 4-week-long differentiation protocol, respectively. Subsequent gene expression analysis showed that the largest adipocyte spheroids (35,000 cells) had the most consistent upregulation of adipogenesis marker *PPARγ* after the 3-week differentiation protocol (Figure S1B). Extending differentiation beyond three weeks did not markedly increase *PPARγ* expression levels in either donor (Figure S1B). Notably, *PPARγ* expression levels in adipocyte spheroids were very similar to those of subcutaneous mature adipocyte fraction cells (MAFs) (donor 3, Table 1, Figure S1B), suggesting that our protocol produces white adipocytes with the physiologically relevant gene expression. Based on these results, we selected 35,000 preadipocytes per spheroid with a 3-week differentiation duration for subsequent experiments.

### Extracellular matrix improves mitochondrial network and respiration

Previous studies have shown that extracellular matrix (ECM) and especially the addition of ECM protein mixtures such as growth-factor reduced Matrigel (GFR-Matrigel) improve lipid droplet morphology and the metabolic phenotype of human white adipocyte spheroids.^11^^,^^16^ Thus, we determined whether GFR-Matrigel also affects mitochondrial metabolism by using adipocyte spheroids derived from the donor 2. After a 3-week differentiation period, the human adipocyte spheroids were stained with MitoTracker, a mitochondrial marker, and analyzed using fluorescence live cell imaging. The results revealed that the scaffold-free adipocyte spheroids had a more dense spheroid structure as well as smaller and more fragmented mitochondria (Figure S2A). In contrast, the GFR-Matrigel embedded adipocyte spheroids exhibited a loose spheroid structure with highly connected and elongated mitochondrial network (Figure S2A). Bright-field images after a 3-week differentiation period verified the morphological differences; the scaffold-free adipocyte spheroids were smaller and more compact, while the GFR-Matrigel embedded adipocyte spheroids were larger with a visible dense core and a fluffy outer rim, as previously reported for other ECM-embedded adipocyte spheroids.^16^^,^^17^^,^^18^

To evaluate how GFR-Matrigel influences mitochondrial function, we established and optimized mitochondrial respiration measurement with the Seahorse Analyzer using adipocyte spheroids derived from donor 2. Interestingly, differentiation of adipocyte spheroids within GFR-Matrigel significantly improved several mitochondrial respiration-related variables, including basal, ATP-linked, and maximal respiration as well as the spare respiratory capacity when compared to scaffold-free differentiated adipocyte spheroids (Figures S2C and S2D). We did not observe significant changes in proton leak (*i.e*., uncoupling) or coupling efficiency (*i.e*., the proportion of mitochondrial electron transport chain activity used to drive basal state ATP synthesis) (Figures S2C and S2D). We also tested how the cell number per (pre)adipocyte spheroid affects the unnormalized oxygen consumption rates (OCRs) values and the reliability of the Seahorse measurements. Notably, unnormalized OCR values above 20 pmol/min have been considered as trustworthy for respiration detection.^19^ Although basal and maximal respiration OCR values for the GFR-Matrigel embedded differentiated adipocyte spheroids containing 15,000 cells were above 20 pmol/min, the scaffold-free undifferentiated preadipocyte spheroids with the same cell number did not yield reliable OCR values (Figure S2E). However, the scaffold-free undifferentiated preadipocyte spheroids with 35,000 cells did demonstrate OCR values above 20 pmol/min (Figure S2E). This suggests that a cell count of 35,000 is required for reliable detection of mitochondrial respiration both in the undifferentiated and differentiated (pre)adipocyte spheroids. Collectively, the embedding of human adipocyte spheroids in GFR-Matrigel seemed to offer an advantageous environment for adipocytes’ mitochondrial network and respiration. As a result, we selected the spheroid formation protocol involving 35,000 preadipocytes embedded in GFR-Matrigel and differentiation in human serum- and PPARγ agonist thiazolidinedione-free medium for three weeks as our gold standard protocol, and all subsequent experiments in this article were conducted using this protocol if not stated otherwise.

### Differentiated adipocyte spheroids recapitulate the subcutaneous white adipocyte phenotype

**Figure 1:**
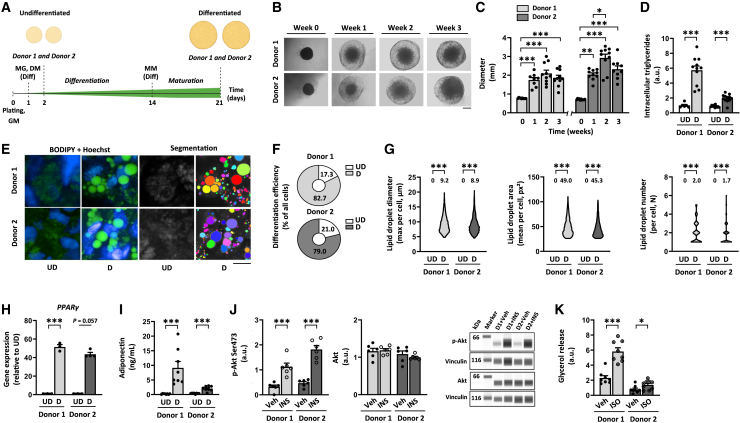
Differentiated human subcutaneous white adipocyte spheroids display a morphological, transcriptional, and metabolic profile typical of mature white adipocytes (A) Schematic presentation of the study design. All experiments were conducted using (pre)adipocyte spheroids derived from donors 1 and 2. GM, growth medium without serum; MG, growth factor reduced-Matrigel; DM, adipocyte differentiation medium; Diff, administration to only differentiated adipocyte spheroids; MM, adipocyte maintenance medium. (B) Selected bright-field images of the adipocyte spheroids from donors 1 and 2 during the 3-week differentiation period. Scale bar = 500 μm (C) Adipocyte spheroid diameter development during the 3-week differentiation period, N = 8–10 per group. (D) Intracellular triglyceride content in undifferentiated (UD) and differentiated (D) (pre)adipocyte spheroids, N = 6–12 per group. (E) Selected representative fluorescence live cell images of lipid droplets (BODIPY, green), nuclei (Hoechst, blue), and the corresponding segmentation masks used for the lipid droplet quantification in differentiated adipocyte spheroids. Mask colors represent individual segmented objects within the imaged area. Scale bar = 20 μm, 40x air objective. Fluorescence images were acquired from a defined zoomed-in region. The same region was used for the mitochondrial images shown in Figure 2, ensuring comparability. Corresponding merged images are presented in Figure S4. (F) Differentiation efficiency in differentiated adipocyte spheroids, N = 4 per group. (G) Lipid droplet maximal diameter, mean area, and number per cell in UD and D (pre)adipocyte spheroids, N = 3–4 (pre)adipocyte spheroids per group. Mean values per group are reported above the violin plots. Note that lipid droplets above the settled threshold were included only from the differentiated adipocyte spheroids. For UD, only cells with values below the set threshold were included. The number of cells analyzed is depicted in the brackets for each analysis as follows: lipid droplet maximal diameter (535–2391 cells/spheroid), lipid droplet mean area (537–2625), and lipid droplet number (534–2153). (H) Gene expression of *PPARγ* in UD and D (pre)adipocyte spheroids, N = 3–4 per group. Data are presented as relative to UD = 1. (I) Media adiponectin concentration from UD and D (pre)adipocyte spheroids, N = 8 per group. (J) Protein content of phosphorylated Akt^Ser473^ and total Akt in response to a 30-min vehicle (Veh, 1x PBS) or 100 nM insulin (INS) administration in differentiated adipocyte spheroids, N = 6 per group. Representative blots are shown on the right. Vinculin was used for data normalization. (K) Glycerol release in response to a 3-h vehicle (Veh, water) or 10 μM isoproterenol (ISO) administration in differentiated adipocyte spheroids, N = 8–9 per group. In (C and D) and (H–K), data are shown as means ± SEM with individual values. In (C) and (J and K), statistical analyses were performed with one-way ANOVA followed by Uncorrected Fisher’s LSD or with Kruskal-Wallis test followed by uncorrected Dunn’s test. In (D) and (F–I), the unpaired *t* test or Mann-Whitney U-test was used. ∗*p* < 0.05, ∗∗*p* < 0.01, and ∗∗∗*p* < 0.001. For related data, see Figures S3A–S3G and S4A.

After the identification of the optimal differentiation protocol for our adipocyte spheroid model, we characterized the morphology, differentiation marker expression, and metabolic properties of the undifferentiated and differentiated (pre)adipocyte spheroids derived from donors 1 and 2 (Figure 1A). Upon morphological analysis, we noticed that the size of the adipocyte spheroids increased during the first week of differentiation (Figures 1B and 1C). Thereafter, the size remained stable for the donor 1, while the adipocyte spheroids derived from the donor 2 showed a slight increase in diameter between week one and two (Figures 1B and 1C). On average, the adipocyte spheroid diameter was 2- to 3-fold higher after 3 weeks, nearly 2 mm, regardless of the cell donor (Figure 1C). Interestingly, total DNA content, a proxy for the cell number, did not significantly differ between the undifferentiated and differentiated (pre)adipocyte spheroids, suggesting that cells did not significantly proliferate during the differentiation (Figure S3A). Despite the rather large size of the differentiated adipocyte spheroids, cryosections did not reveal signs of a necrotic center (Figure S3B). In the cryosections, the empty spaces, considered as remnants of lipid droplets and GFR-Matrigel, were abundant throughout the adipocyte spheroids, suggesting the presence of differentiated adipocytes also in the core of the adipocyte spheroids (Figure S3B). In line, the differentiated adipocyte spheroids from either donor did not demonstrate any significant cell death based on the low lactate dehydrogenase (LDH) enzyme activity in the cell culture medium (<20 U/L vs. ≤ 245 U/L expected in human serum based on a previous report^20^ and the manufacturer’s instructions, Figure S3C).

To validate the level of differentiation and maturation of the adipocytes, we measured several adipogenic markers. The analysis of intracellular triglyceride content showed a significant accumulation of lipids into the differentiated adipocyte spheroids in comparison to the respective undifferentiated preadipocyte spheroids cultured for 2 days (Figure 1D). The increase in stored triglycerides was higher for the adipocyte spheroids derived from donor 1 (Figure 1D). Next, we used fluorescence live cell imaging with a BODIPY lipid marker to analyze lipid droplet morphology and number in non-fixed human adipocyte spheroids. This technique enabled us to examine a large cell population in the outer layer of the adipocyte spheroids. Only in the differentiated adipocyte spheroids, there was an appearance of adipocytes with lipid droplets, and these adipocytes demonstrated uni- and multilocular lipid droplet morphology (Figures 1E and S4A). The proportion of lipid-containing cells with unilocular lipid droplets was near 50% (donor 1; mean 47.0 ± SEM 5.7% and donor 2; mean 54.3 ± SEM 3.8%) of the analyzed cells per spheroid. The differentiation efficiency, evaluated as the ratio of differentiated adipocytes to the total number of imaged cells per differentiated adipocyte spheroid, was high, nearly 80%, regardless of the donor (Figure 1F). For both donors, the average diameter of the largest lipid droplet per cell (including both uni- and multilocular droplets) was ∼9 μm for the differentiated adipocyte spheroids (Figure 1G). Typically, the diameter range varied between 5 and 20 μm (Figure 1G). On average, the mean lipid droplet area (∼50 px^2^) and lipid droplet number (∼2) per cell were similar in the differentiated adipocyte spheroids from both donors (Figure 1G). These changes in lipid deposition and lipid droplet morphology were accompanied by the upregulated gene expression of common adipogenesis markers, including *PPARγ* (Figure 1H), fatty acid synthase (*FASN*), hormone sensitive lipase (*HSL*), adiponectin (*ADIPOQ*), and leptin (*LEPTIN*) (Figure S3D). The changes in gene expression profile were greater in the adipocyte spheroids derived from donor 1 (Figures 1H and S3D). The gene expression of a beiging/browning marker, *UCP1*, increased in the differentiated adipocyte spheroids in comparison to the respective undifferentiated preadipocyte spheroids, especially in donor 1 (Figure S3E). However, in comparison to scWAT and subcutaneous MAF pool from multiple donors (donors 4–9, Table 1), the *UCP1* expression in adipocyte spheroids remained within the normal physiological range (Figure S3E). In conclusion, our adipocyte spheroids fulfilled the criteria for efficient adipogenic differentiation, including elevated lipid accumulation, the formation of white adipocytes with unilocular lipid droplets, and an adipogenic expression profile consistent with mature white adipocytes.

Next, we analyzed adipokine secretion capacity by quantifying medium adiponectin and leptin concentration in our model. The differentiated adipocyte spheroids from both donors had significantly higher adiponectin and leptin secretion as compared to the respective undifferentiated preadipocyte spheroids (Figures 1I and S3F). Interestingly, we noticed that the adiponectin and leptin secretion capacity was affected by the passaging of predipocytes used to generate the adipocyte spheroids. More precisely, a lower passage number (two to three vs. six) of predipocytes markedly facilitated adiponectin and leptin secretion to the cell culture medium even in the adipocyte spheroids with a slightly lower cell number than in our golden standard protocol (32,500 vs. 35,000 cells per spheroid) (Figure S3G). In conclusion, adipokine secretion in the 3D model increased with differentiation and was modulated by preadipocyte passage number, indicating a physiological functionality of the adipocyte spheroid model.

To validate our 3D culture model, we compared its adipogenic and endocrine characteristics to a 2D monolayer human white adipocyte differentiation protocol published to generate the whitest adipocyte phenotype to date, despite predominantly multilocular lipid droplets.^21^ Our 3D differentiated adipocyte spheroids exhibited significantly higher expression of the key adipogenic markers involved in lipid metabolism such as *PPARγ, FASN,* and *HSL* (Figure S3H). Interestingly, the adipokine-related adipogenic markers *ADIPOQ* and *LEPTIN* showed greater expression in the 2D monolayer cultured adipocytes (Figure S3H), and *UCP1* expression peaked in donor 1’s 2D adipocytes, also exceeding that of scWAT and pooled subcutaneous MAF samples (Figure S3I). Functionally, our 3D adipocyte spheroids secreted equal or higher adiponectin levels compared to the 2D model, depending on the donor, while leptin secretion remained limited in the spheroids from both donors (Figure S3J). Together, these results support the physiological relevance of our 3D spheroid model in recapitulating white adipocyte differentiation and adipokine secretion, particularly the robust release of adiponectin compared to leptin.

Lastly, to study the responsiveness of our spheroids to hormonal signals, we administered the differentiated adipocyte spheroids with insulin or isoproterenol to investigate their downstream protein phosphorylation and lipolytic responses, respectively. Our results showed that independent of the donor characteristics, insulin stimulation greatly increased the phosphorylation of Akt^Ser473^ in the differentiated adipocyte spheroids, while total Akt remained unchanged (Figure 1J). In addition, the differentiated adipocyte spheroids from both donors responded to isoproterenol by secreting glycerol into the medium ≥2-fold in comparison to basal, non-stimulated conditions (Figure 1K). That being said, donor 1 had a greater response to the isoproterenol administration (Figure 1K).

Together, these results demonstrated that our adipocyte spheroids exhibit morphological, transcriptional, metabolic, and functional profiles typical of *in vivo* subcutaneous white adipocytes.^3^

### Differentiated adipocyte spheroids exhibit increased mitochondrial respiration and content

**Figure 2:**
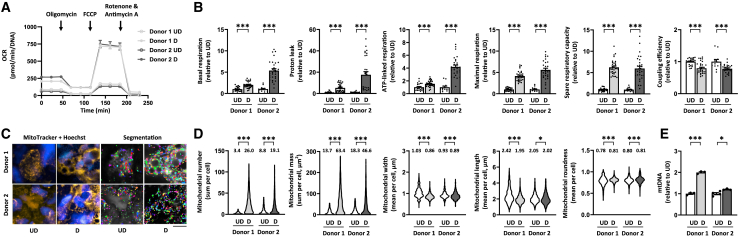
Differentiation of human subcutaneous white adipocyte spheroids increases mitochondrial respiration, biogenesis, and fragmentation (A) Mitochondrial respiration analysis in undifferentiated (UD) and differentiated (D) (pre)adipocyte spheroids derived from donors 1 and 2. Representative OCR values were normalized to total DNA amount. (B) Parameters of mitochondrial respiration, including basal respiration, proton leak, ATP-linked respiration, maximal respiration, spare respiratory capacity, and coupling efficiency in UD and D (pre)adipocyte spheroids,N = 13–32 per group. (C) Selected representative fluorescence live cell images of the mitochondria (MitoTracker, yellow), nuclei (Hoechst, blue), and the corresponding segmentation masks used for the quantification of mitochondrial parameters in undifferentiated and differentiated (pre)adipocyte spheroids from donors 1 and 2. Mask colors represent individual segmented objects within the imaged area. Scale bar = 20 μm, magnification is 40× air objective. Fluorescence images were acquired from the same zoomed-in region as in Figure 1 to ensure comparability between figures. Corresponding merged images are presented in Figure S4. (D) Quantification of mitochondrial number, mass, width, length, and roundness based on the fluorescence live cell imaging, N = 3–4 (pre)adipocyte spheroids per group. Mean values per group are reported above the violin plots. The number of cells analyzed is depicted in the brackets for each analysis as follows: mitochondrial number (648–2960 cells/spheroid), mitochondrial mass (713–2966), mitochondrial width (764–3176), mitochondrial length (747–3184), and mitochondrial roundness (788–3185). (E) MtDNA amount expressed per nuclear genome in UD and D (pre)adipocyte spheroids, N = 3–4 per group. In (B) and (E), data are presented as relative to UD = 1. In (A and B) and (E), data are shown as means ± SEM with individual values. In (B and D), the unpaired *t* test or the Mann-Whitney U-test was used. ∗*p* < 0.05 and ∗∗∗*p* < 0.001. For related data, see Figures S3K and S4A.

Given that mitochondria are known to be crucial during adipogenesis to fulfill the energy needs of differentiating cells and to support adipokine secretion,^22^ we next asked whether our adipocyte spheroid model can be exploited to detect the remodeling of mitochondrial metabolism that occurs during adipocyte differentiation.^23^^,^^24^ We first used respirometry to compare the undifferentiated and differentiated (pre)adipocyte spheroids from both donors. We found OCR to be in the detectable range with the chosen adipocyte spheroid size (Figure S3K). The differentiated adipocyte spheroids from both donors showed significantly elevated basal respiration, proton leak, ATP-linked respiration, maximal respiration, and spare respiratory capacity in comparison to the respective undifferentiated preadipocyte spheroids (Figures 2A and 2B). Coupling efficiency was significantly reduced in the differentiated adipocyte spheroids from both donors in comparison to the undifferentiated preadipocyte spheroids (Figure 2B), suggesting less efficient mitochondrial respiration after the 3-week differentiation.

To assess changes in mitochondrial content and morphology upon adipocyte spheroid differentiation, we employed fluorescence live cell imaging, focusing on the outer layer of adipocytes of the differentiated spheroids (Figures 2C and S4A). Upon differentiation, mitochondrial number and mass per cell were significantly increased (Figure 2D), with donor 1 demonstrating a greater increase in these two variables. Mitochondrial width and length slightly decreased upon differentiation, while roundness increased, particularly in donor 1, possibly leading to the formation of smaller, more fragmented mitochondria (Figure 2D). In line with the imaging data, the amount of mitochondrial DNA (mtDNA) per nuclear genome was significantly increased in the differentiated adipocyte spheroids derived from both donors in comparison to the respective undifferentiated preadipocyte spheroids (Figure 2E). The increase in mtDNA amount was higher in the adipocyte spheroids derived from donor 1.

Taken together, our results demonstrated that adipocyte spheroid differentiation induced an increase in mitochondrial respiration and biogenesis, as expected based on the growing body of literature.^23^^,^^24^ These findings highlight the suitability and usability of our human adipocyte spheroid model for reliable analysis of mitochondrial metabolism related variables in physiological conditions.

### Lipid mixture administration promotes lipid accumulation and impairs metabolic homeostasis and mitochondrial function

**Figure 3:**
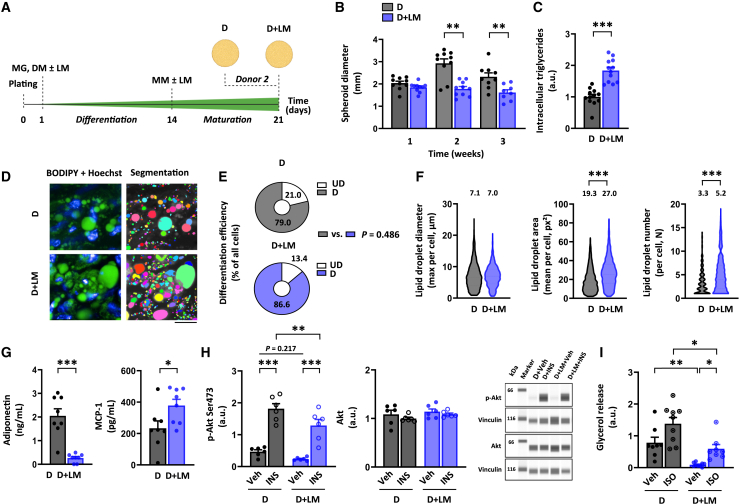
Lipid mixture administration increases lipid accumulation and impairs endocrine function and hormonal responsiveness (A) Schematic presentation of the study design. Only differentiated adipocyte spheroids derived from donor 2 were used in these experiments. (B) Adipocyte spheroid diameter development without (D) or with lipid mixture (LM) administration (D + LM) during the 3-week differentiation period, N = 8–10 per group. (C) Intracellular triglyceride content in D and D + LM adipocyte spheroids, N = 12 per group. Data are presented as relative to D = 1. (D) Selected representative fluorescence live cell images of lipid droplets (BODIPY, green), nuclei (Hoechst, blue), and the corresponding segmentation masks used for the lipid droplet quantification in D and D + LM adipocyte spheroids. Mask colors represent individual segmented objects within the imaged area. Scale bar = 20 μm, magnification 40× air objective. Fluorescence images were acquired from a defined zoomed-in region. The same region was used for the mitochondrial images shown in Figure 4, ensuring comparability. Corresponding merged images are presented in Figure S4. (E) Differentiation efficiency in D and D + LM adipocyte spheroids, N = 4 per group. (F) Lipid droplet maximal diameter, mean area, and number in D and D + LM adipocyte spheroids, N = 4 per adipocyte spheroid group. Mean values per group are reported above the violin plots. The number of cells analyzed is depicted in the brackets for each analysis as follows: lipid droplet maximal diameter (2372–4495 cells/spheroid), lipid droplet mean area (3060–4377), and lipid droplet number (1832–4044). (G) Adiponectin and monocyte chemoattractant protein 1 (MCP-1) concentrations from D and D + LM adipocyte spheroids, N = 8 per group. (H) Protein content of phosphorylated Akt^Ser473^ and total Akt in response to a 30-min vehicle (Veh, 1x PBS) or 100 nM insulin (INS) administration in D and D + LM adipocyte spheroids, N = 6 per group. Representative blots are shown on the right. Vinculin was used for data normalization. (I) Glycerol release in response to a 3-h vehicle (Veh, water) or 10 μM isoproterenol (ISO) administration in D and D + LM adipocyte spheroids, N = 8–9 per group. In (B and C) and (G–I), data are shown as means ± SEM with individual values. In (B and C) and (E–G), an unpaired *t* test or Mann-Whitney U-test was used. In (H and I), one-way ANOVA followed by uncorrected Fisher’s LSD or Kruskal–Wallis test followed by uncorrected Dunn’s test was used. ∗*p* < 0.05, ∗∗*p* < 0.01, and ∗∗∗*p* < 0.001. For related data, see Figures S4B and S5A–S5D.

To evaluate whether we could mimic mitochondrial dysfunction and metabolic complications in adipocytes typically induced by a nutrient-based energy surplus during obesity,^3^ we added a lipid mixture (LM) to the medium of adipocyte spheroids derived from donor 2 with a higher BMI during the 3-week differentiation protocol (Table 1; Figure 3A). We selected a commercial LM that contains both unsaturated and saturated fatty acids as well as cholesterol because everyday foods normally contain a mixture of these lipid classes.^25^^,^^26^

Starting in week 2 of LM administration, the adipocyte spheroid diameter remained ∼50% lower as compared to the LM-free controls, with a diameter around 1.5 mm per spheroid at the end of the differentiation (Figures 3B and S5A). Given that total DNA content decreased for the adipocyte spheroids after LM administration in comparison to the LM-free adipocyte spheroids (Figure S5B), the smaller adipocyte spheroid size could be due to the lower cell proliferation and/or more efficient packing of lipids into larger lipid droplets. Notably, LM administration did not promote cell death compared to the LM-free counterparts as evaluated based on LDH activity in the medium (Figure S5C).

As expected, intracellular triglyceride content in adipocyte spheroids was significantly increased in response to LM administration during differentiation (Figure 3C). Based on the fluorescence live cell imaging (Figures 3D and S4B) and image analysis, we found that the differentiation efficiency did not significantly differ between the LM-administered and LM-free counterparts in the imaged areas (Figure 3E). In the LM-administered adipocyte spheroids, the maximum lipid droplet diameter per cell was comparable to that of LM-free counterparts, but both mean lipid droplet area and number per cell were significantly elevated (Figure 3F). LM administration was associated with a slight decline in the proportion of unilocular cells, but it did not reach statistical significance (D; mean 29.0 ± SEM 6.0% and D + LM; mean 19.3 ± SEM 0.8%, *p* = 0.155). Importantly, the expression of *PPARγ* and *UCP1* did not significantly differ between the LM-administered and LM-free counterparts (Figure S5D). In conclusion, LM administration increased lipid accumulation and promoted the formation and enlargement of lipid droplets, without affecting adipocyte spheroid differentiation or beiging.

In comparison to the LM-free counterparts, the LM-administered adipocyte spheroids had significantly reduced adiponectin secretion (Figure 3G). Due to the limited leptin secretion observed, we concentrated our adipokine analyses on adiponectin. In contrast, the secretion of proinflammatory cytokine MCP-1, which is known to be induced in scWAT in obesity,^27^ was significantly enhanced after LM administration (Figure 3G). Besides impairing adiponectin secretion, LM-induced lipid accumulation also affected their responsiveness to hormonal stimuli. Namely, insulin-stimulated phosphorylated Akt^Ser473^ levels were lower after LM administration, suggesting an impairment of insulin signaling (Figure 3H). Total Akt remained unchanged in all conditions (Figure 3H). LM administration also significantly reduced glycerol release in basal and isoproterenol stimulated conditions (Fig ure 3I). Thus, LM-administration impaired adipocyte spheroid endocrine secretion and hormonal responsiveness.

**Figure 4:**
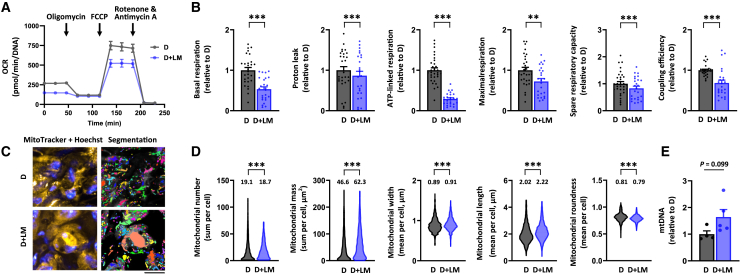
Lipid mixture administration results in mitochondrial bioenergetic defect. Only differentiated adipocyte spheroids derived from donor 2 were used in these experiments (A) Mitochondrial respiration analyses in differentiated adipocyte spheroids without (D) and with lipid mixture (D + LM) administration. Representative OCR values were normalized to the total DNA amount. (B) Parameters of mitochondrial respiration, including basal respiration, proton leak, ATP-linked respiration, maximal respiration, spare respiratory capacity, and coupling efficiency in D and D + LM adipocyte spheroids, N = 25–31 per group. (C) Selected representative fluorescence live cell imaging of the mitochondria (MitoTracker, yellow), nuclei (Hoechst, blue), and the corresponding segmentation masks used for the quantification of mitochondrial parameters in D and D + LM adipocyte spheroids. Mask colors represent individual segmented objects within the imaged area. Scale bar = 20 μm, magnification 40× air objective. Fluorescence images were acquired from the same zoomed-in region as in Figure 3 to ensure comparability between figures. Corresponding merged images are presented in Figure S4. (D) Quantification of mitochondrial number, mass, width, length, and roundness based on the fluorescence live cell imaging. Mean values per group are reported above the violin plots. N = 4 adipocyte spheroids per group. The number of cells analyzed is depicted in the brackets for each analysis as follows: mitochondrial number (1759–2473 cells/spheroid), mitochondrial mass (2089–2447), mitochondrial width (2163–2671), mitochondrial length (2157–2659), and mitochondrial roundness (2209–2737). (E) MtDNA amount expressed per nuclear genome in D and D + LM adipocyte spheroids, N = 4 per group. In (B) and (E), data are presented as relative to D = 1. In (A and B) and (E), data are shown as means ± SEM with individual values. In (B and D), the unpaired *t* test or the Mann-Whitney U-test was used. ∗∗*p* < 0.01 and ∗∗∗*p* < 0.001. For related data, see Figure S4B.

The Seahorse mitochondrial respirometry assay revealed that LM administration during differentiation significantly reduced basal respiration, proton leak, ATP-linked respiration, maximal respiration, and spare respiratory capacity in the adipocyte spheroids compared to their LM-free counterparts (Figures 4A and 4B). Coupling efficiency was also markedly reduced, indicating mitochondrial inefficiency following chronic exposure to LM (Figure 4B). Fluorescence live cell imaging (Figures 4C and S4B) and image analysis showed a slight decrease in mitochondrial number but a significant increase in mitochondrial mass compared to the LM-free counterparts (Figure 4D). LM administration, while reducing roundness, increased mitochondrial width and length, suggesting possibly larger and more elongated mitochondria (Figure 4D). Additionally, mtDNA content showed an increasing trend after LM administration (Figure 4E). These findings suggest that LM administration induces mitochondrial respiration defects alongside increased mitochondrial biogenesis.

Collectively, LM administration during adipocyte spheroid differentiation promoted lipid accumulation, along with lipid droplet biosynthesis and enlargement, and adipocyte dysfunction. These alterations were accompanied by mitochondrial bioenergetic defects and a compensatory increase in mitochondrial biogenesis. We conclude that our adipocyte spheroid model with LM administration can be exploited to mimic and study nutrient-induced obesity-associated mitochondrial dysfunction and metabolic disturbances.

### Rosiglitazone alleviates lipid mixture administration-induced disturbances in mitochondrial metabolism

**Figure 5:**
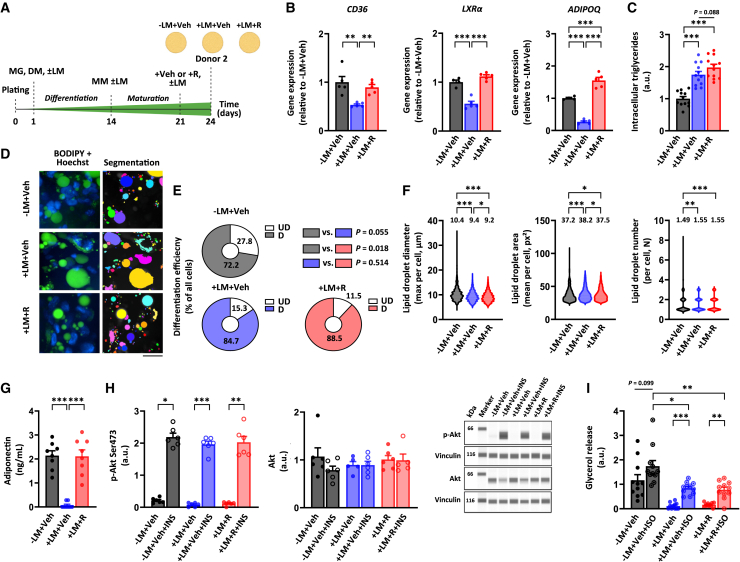
Rosiglitazone treatment increases PPARγ transcriptional activity and restores adipokine secretion with a lesser effect on hormonal responsiveness (A) Schematic presentation of the study design. Only differentiated adipocyte spheroids derived from donor 2 were used in these experiments. (B) Gene expression of *CD36*, *LXRα*, and *ADIPOQ* in differentiated adipocyte spheroids administered without lipid mixture (LM) but with vehicle (−LM + Veh), with LM and vehicle (+LM + Veh) or with LM and 1 μM rosiglitazone (+LM + R) for 72 h post-differentiation, N = 4–5 per group. Data are presented as relative to −LM + Veh = 1. (C) Intracellular triglyceride content in adipocyte spheroids between the groups, N = 12 per group. (D) Selected representative fluorescence live cell images of lipid droplets (BODIPY, green), nuclei (Hoechst), and the corresponding segmentation masks used for the lipid droplet quantification in adipocyte spheroids from the different groups. Mask colors represent individual segmented objects within the imaged area. Scale bar = 20 μm, magnification 40× air objective. Fluorescence images were acquired from a defined zoomed-in region. The same region was used for the mitochondrial images shown in Figure 6, ensuring comparability. Corresponding merged images are presented in Figure S4. (E) Differentiation efficiency in adipocyte spheroids among the groups, N = 4 per group. The results of statistical analyses between the groups are presented on the top right. (F) Lipid droplet maximal diameter, mean area, and number in adipocyte spheroids between the groups, N = 4 adipocyte spheroids per group. Mean values per group are reported above the violin plots. The number of cells analyzed per group is depicted in the brackets for each analysis as follows: lipid droplet maximal diameter (882–2234 cells/spheroid), lipid droplet mean area (835–2085), and lipid droplet number (832–2064). (G) Adiponectin concentration between the groups, N = 8 per group. (H) Protein content of phosphorylated Akt^Ser473^ and total Akt in response to a 30-min vehicle (Veh, 1x PBS) or 100 nM insulin (INS) administration in adipocyte spheroids between the groups, N = 6 per group. Representative blots are shown on the right. Vinculin was used for data normalization. (I) Glycerol release in response to a 3-h vehicle (Veh, water) or 10 μM isoproterenol (ISO) administration in adipocyte spheroids between the groups, N = 11 per group. In (B and C) and (G–I), data are shown as means ± SEM with individual values. In (B and C) and (E–I), one-way ANOVA followed by uncorrected Fisher’s LSD or Kruskal–Wallis test followed by uncorrected Dunn’s test was used. ∗*p* < 0.05, ∗∗*p* < 0.01, and ∗∗∗*p* < 0.001. For related data, see Figures S4C and S5E.

After demonstrating that our adipocyte spheroids serve as a viable model for studying adipocyte mitochondrial metabolism also under pathophysiological conditions, we next aimed to determine whether the adverse effects of LM administration on mitochondrial function and metabolic homeostasis could be mitigated or even reversed pharmacologically. For this purpose, we chose to use the antidiabetic drug rosiglitazone, which activates PPARγ-controlled transcription.^28^ In these experiments, LM-administered adipocyte spheroids derived from donor 2 were treated with rosiglitazone at a concentration of 1 μM for 72 h after first undergoing the 3-week differentiation protocol (+LM + R, Figure 5A). As PPARγ activation is known to upregulate the transcription of its downstream target genes, such as fatty acid translocase (*FAT*, also known as *CD36*), liver X receptor α (*LXRα*), and adiponectin (*ADIPOQ*),^28^^,^^29^ we used the expression of these genes as a readout for rosiglitazone-induced PPARγ activation. We observed that LM administration combined with vehicle treatment (+LM + Veh) significantly downregulated the expression of *CD36*, *LXRα,* and *ADIPOQ* in comparison to vehicle treatment without LM (-LM + Veh), which was rescued by rosiglitazone treatment (Figure 5B). *PPARγ* expression was significantly downregulated by LM administration, both in combination with vehicle and with rosiglitazone (Figure S5E). Notably, *UCP1* expression remained unchanged after rosiglitazone treatment together with LM (Figure S5E). Collectively, these results confirmed that rosiglitazone treatment activated PPARγ transcriptional activity in our differentiated adipocyte spheroids without affecting adipocyte beiging.

Rosiglitazone treatment did not significantly promote intracellular triglyceride accumulation into LM administered adipocyte spheroids from donor 2 as compared to vehicle (Figure 5C). Based on the fluorescence live cell imaging (Figures 5D and S4C) and image analysis, we found differentiation efficiency to be enhanced in both vehicle and rosiglitazone-treated LM-administered adipocyte spheroids compared to their LM-free counterparts (Figure 5E). However, rosiglitazone did not significantly impact the differentiation efficiency within the LM-administered adipocyte spheroids (Figure 5E). Rosiglitazone exacerbated the LM-induced decline in the maximal diameter of lipid droplets, but it mitigated the LM-induced increase in the mean lipid droplet area (Figure 5F). In turn, rosiglitazone did not influence the increase in lipid droplet number caused by LM administration (Figure 5F). The fraction of unilocular cells in adipocyte spheroids was not significantly affected by rosiglitazone treatment as compared to vehicle (−LM + Veh; mean 69.0 ± SEM 10.6%, +LM + Veh; mean 57.3 ± SEM 2.8%, and +LM+R; mean 56.8 ± SEM 4.0%, +LM + Veh vs. +LM + R, *p* = 0.959). These results suggest that rosiglitazone may promote the shrinkage of the lipid droplet size in adipocyte spheroids.

Interestingly, rosiglitazone treatment completely restored the LM-induced decrease in adiponectin secretion from the adipocyte spheroids derived from donor 2 (Figure 5G). In contrast, basal and insulin-stimulated Akt^Ser473^ phosphorylation were not markedly affected by rosiglitazone treatment upon LM administration (Figure 5H). In addition, total Akt remained unchanged in all analyzed conditions (Figure 5H). In line with the insulin signaling-related results, rosiglitazone did not alleviate the LM-induced impairment in basal and isoproterenol-stimulated glycerol release in adipocyte spheroids (Figure 5I).

**Figure 6:**
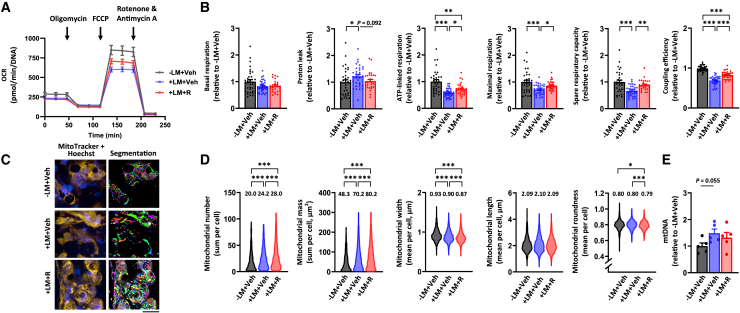
Rosiglitazone treatment alleviates lipid mixture administration-induced disturbances in mitochondrial metabolism Only differentiated adipocyte spheroids derived from donor 2 were used in these experiments. (A) Mitochondrial respiration analyses in differentiated adipocyte spheroids administered without lipid mixture (LM) but with vehicle (−LM + Veh), with LM and vehicle (+LM + Veh), or with LM and 1 μM rosiglitazone (+LM + R) for 72h post differentiation. Representative OCR values were normalized to the total DNA amount. (B) Parameters of mitochondrial respiration, including basal respiration, proton leak, ATP-linked respiration, maximal respiration, spare respiratory capacity, and coupling efficiency in adipocyte spheroids between the groups,N = 28–33 per group. Selected representative fluorescence live cell images of mitochondria (MitoTracker, yellow), nuclei (Hoechst), and the corresponding segmentation masks used for the quantification of mitochondrial parameters in adipocyte spheroids between the groups. Mask colors represent individual segmented objects within the imaged area. Scale bar = 20 μm, magnification 40× air objective. Fluorescence images were acquired from the same zoomed-in region as in Figure 5 to ensure comparability between figures. Corresponding merged images are presented in Figure S4. (D) Quantification of mitochondrial number, mass, width, length, and roundness was analyzed based on the imaging analysis. Note that for clarity, *y* axis in mitochondrial roundness does not start from zero. Mean values per group are reported above the violin plots, N = 4 adipocyte spheroids per group. The number of cells analyzed per group is depicted in the brackets for each analysis as follows: mitochondrial number (1653–3293 cells/spheroid), mitochondrial mass (1654–3370), mitochondrial width (1790–3414), mitochondrial length (1748–3415), and mitochondrial roundness (1807–3476). (E) MtDNA amount expressed per nuclear genome in adipocyte spheroids, N = 5 per group. In (B) and (E), data are presented as relative to −LM + Veh = 1. In (A and B) and (E), data are shown as means ± SEM with individual values. In (B and D), one-way ANOVA followed by uncorrected Fisher’s LSD or Kruskal-Wallis followed by uncorrected Dunn’s test was used. ∗*p* < 0.05, ∗∗*p* < 0.01 and ∗∗∗*p* < 0.001. For related data, see Figure S4C.

Notably, rosiglitazone treatment did not improve basal respiration, although it partially corrected the LM-induced defects in ATP-linked respiration, maximal respiration, and spare respiratory capacity in the adipocyte spheroids derived from donor 2 (Figures 6A and 6B). In addition, rosiglitazone tended to decrease the LM-induced proton leak with the concomitant increase in coupling efficiency, thus suggesting improved ATP production (Figure 6B). Fluorescence live cell imaging (Figures 6C and S4C) and subsequent image analysis showed that rosiglitazone treatment further amplified the LM-induced increase in mitochondrial number and mass (Figure 6D). Additionally, while rosiglitazone did not alter mitochondrial length, it further reduced the LM-induced decrease in width, resulting in slightly less round and probably a bit more elongated mitochondria (Figure 6D). However, rosiglitazone did not affect mtDNA amount (Figure 6E). Collectively, rosiglitazone likely improved mitochondrial bioenergetics, biogenesis, and dynamics post-differentiation in adipocyte spheroids.

As a whole, our results demonstrated that rosiglitazone treatment increased PPARγ transcriptional activity, which was followed by first-line improvements in mitochondrial metabolism, adiponectin secretion, and lipid droplet size. Our findings underscore that our adipocyte spheroid model has the potential to be used in pharmacological studies aimed at improving mitochondrial activity, metabolic homeostasis, and lipid droplet remodeling in human subcutaneous adipocytes.

## Discussion

Accumulating evidence suggests that human adipocyte spheroid models offer more physiologically relevant *in vitro* cultures than traditional 2D monolayer cultures for adipocyte studies.^9^^,^^11^^,^^12^ However, current adipocyte spheroid models have not been optimized for studying mitochondrial metabolism. Given the crucial role of scWAT mitochondrial dysfunction in the development of obesity-related metabolic complications, there is a clear need to explore adipocyte mitochondrial biology and pharmacological modulators in a suitable model. In this study, we present a novel 3D *in vitro* model of human subcutaneous adipocytes to investigate mitochondrial related variables. Our findings demonstrate that this new model facilitates the study of mitochondria during adipogenic differentiation, upon lipid overload-induced mitochondrial dysfunction, and the effects of mitochondria-targeting compounds.

At present, several human 3D adipocyte models exist, utilizing different cell culture techniques and protocols, with or without ECM scaffolds.^9^^,^^11^^,^^12^ Our model builds on this existing knowledge by using human subcutaneous preadipocytes, ultra-low attachment plates, and a GFR-Matrigel scaffold. However, our model differs notably from the other models by employing human serum during the preadipocyte expansion, a shorter 3-week differentiation period, and a larger adipocyte spheroid size (35,000 cells), the latter of which allows more robust detection of mitochondria-related parameters. As summarized in Table S1, among selected published human adipocyte spheroid models using ultra-low attachment plates, our approach is unique in investigating mitochondrial metabolism in differentiated adipocytes, particularly those embedded in GFR-Matrigel. This ECM scaffold was integrated into the protocol based on our novel finding that it promoted mitochondrial network formation and enhanced mitochondrial respiration, likely by providing a less compact, more oxygen-permeable environment. Consistently, the type of plate coating has been shown to modulate mitochondrial function in 2D cultured rat cardiac myocytes.^30^ These findings highlight how ECM scaffold or biomaterial substrate type can directly influence mitochondrial metabolism, which is an important consideration when engineering *in vitro* human adipocyte models.

By using our newly established model, we observed adipogenesis-induced mitochondrial metabolic remodeling in differentiated adipocyte spheroids, including increased mitochondrial biogenesis and a metabolic shift toward a more oxidative energy profile, as seen in monolayer cultures.^22^^,^^23^^,^^24^ Despite this, the mitochondria became more fragmented and less efficient by the end of differentiation. At first glance, these findings may seem surprising. However, they align with previous mouse adipocyte studies, which show that mitochondrial respiration is initially tightly coupled to ATP synthesis during adipogenesis but becomes uncoupled as adipocytes mature, focusing more on fat storage.^31^ Overall, our model is the first to enable comprehensive analysis of mitochondria-related variables under (patho)physiological conditions, thereby advancing human adipocyte spheroid research.

The existing 3D adipocyte models address many limitations of monolayer cultures, showing improved differentiation and better metabolic functionality.^11^^,^^12^ In our adipocyte spheroid model, we achieved a high differentiation efficiency of nearly 80%, compared to 20–50% in monolayer cultures.^21^ Successful differentiation was confirmed by increased lipid deposition and high expression of adipogenic genes (*PPARγ*, *FASN* and *HSL*) compared to the published 2D protocol,^21^ consistent with previous 3D models.^9^^,^^11^^,^^12^ Our adipocyte spheroids exhibited typical features of mature white adipocytes, including low *UCP1* expression and rigid responses to insulin and β-adrenergic stimuli. Endocrine functionality was evidenced by the secretion of adiponectin and leptin following differentiation, although leptin secretion remained very limited. Compared to the 2D monolayer cultures, adiponectin secretion was equal or higher in our 3D adipocyte spheroids, despite lower *ADIPOQ* expression, whereas leptin secretion and *LEPTIN* expression were reduced in our 3D model. These findings align with reports of enhanced adiponectin but restricted leptin release in 3D human adipocyte cultures.^16^^,^^32^ Notably, vascularized adipocyte spheroids have shown so far the most robust adipokine secretion,^16^ thus suggesting the importance of endothelial cells for this function. These results highlight the complex regulation of adipokine secretion, influenced possibly by microenvironmental cues and factors beyond adipogenesis efficiency. Interestingly, higher passage numbers (six) reduced adiponectin and leptin secretion nearly ten-to 3-fold compared to lower passages (two to three), respectively. This phenomenon may be related to lower differentiation efficiency, a lower overall degree of differentiation upon passaging, elevated cellular senescence, or even batch effects independent of passage number. Importantly, the adipocyte spheroids with low passage number secreted adiponectin and leptin to the medium at levels comparable to those observed in human plasma.^33^ In line with the previous knowledge on human adipose stem cell function,^34^ donor variability also affected differentiation, as adipocyte spheroids derived from donor 2 showed a less pronounced adipogenic phenotype than the donor 1. Taken together, our findings demonstrate that our 3D culture model of adipocyte spheroids offers a physiologically relevant and efficient *in vitro* platform for studying the key aspects of human mature white adipocyte functions, while also highlighting the influence of the microenvironment and local signaling networks on adipokine secretion.

The 3D adipocyte cultures also display a higher proportion of unilocular lipid droplets than in monolayer cultures. In our model, quantification revealed that 30–50% of adipocytes in the outer layer of the spheroids contained unilocular lipid droplets. Not surprisingly, based on the fluorescence live cell imaging, the average maximal lipid droplet diameter (9 μm) in our adipocyte spheroids was smaller than *in vivo* subcutaneous white adipocytes (20–150 μm)^35^ but comparable to other similar 3D adipocyte models,^15^^,^^16^ where the typical droplet diameter has been around 10 μm. Nonetheless, differences in imaging techniques and analysis methods make direct comparison with previously published adipocyte spheroid cultures challenging. For example, the present study used live imaging to quantify all imaged cells (thousands), whereas previous studies^15^^,^^16^ employed fixed cell imaging with a maximum of a few hundred cells. So far, the largest lipid droplets (20–40 μm) have been seen in vascularized adipocyte spheroids,^16^ suggesting that the absence of endothelial cells in our model may explain the slightly smaller lipid droplet size. As the cell composition in 3D cultures seems to greatly influence adipocyte lipid droplet maturation, this calls for a need of the development of *in vitro* culture models in which all major cellular components of scWAT are present. Overall, a significant portion of the adipocytes in our adipocyte spheroids exhibit unilocular lipid droplets, with the size comparable to that observed in most other 3D models of adipocytes.

Administration of fatty acids or triglycerides has been shown to induce lipid deposition and metabolic complications in human adipocytes in 3D culture models.^15^^,^^16^^,^^36^ Consistent with these findings, the addition of LM (a mixture of unsaturated and saturated fatty acids as well as cholesterol) increased the lipid droplet mean size in our adipocyte spheroids and likely stimulated the formation of new lipid droplets, as evidenced by a higher lipid droplet number per cell. While the proportion of unilocular lipid droplets did not significantly change, the LM-induced lipid droplet formation likely occurred in multilocular adipocytes. These changes in lipid droplet morphology were linked to early inflammatory signs, such as elevated levels of the pro-inflammatory cytokine MCP-1, as well as impaired insulin signaling, lipolysis, and endocrine failure. Notably, LM administration during adipogenic differentiation led to a significant decline in mitochondrial respiration variables, despite increased mitochondrial mass and fusion. This suggests that LM administration may trigger mitochondrial biogenesis and fusion in response to functional decline, similar to compensatory mechanisms observed in mitochondrial disorders.^37^ Given that decreased mitochondrial respiration, biogenesis, and fusion are also seen in obesity *in vivo*,^3^ our *in vitro* results indicate that mitochondrial respiration failure may be an early defect associated with lipid overload. The relationship between elevated MCP-1 levels and mitochondrial dysfunction warrants further investigation to determine causality, as cells with defective mitochondria can produce pro-inflammatory cytokines.^38^ Overall, these results highlight the relevance of our human adipocyte spheroids as a model system that mimics human subcutaneous white adipocytes in obesity-related conditions and provides a platform for studying the molecular mechanisms of adipocytes’ mitochondrial dysfunction.

As scWAT mitochondrial dysfunction is involved in the pathogenesis of obesity,^3^ pharmacological compounds that improve adipocytes’ mitochondrial function represent an important treatment prospect for obesity. Rosiglitazone has been shown to enhance mitochondrial respiration, biogenesis, and fusion in human white adipocytes,^39^^,^^40^ but its clinical use is limited due to the side effects such as heart failure, weight gain, and peripheral oedema.^41^ In our adipocyte spheroid model, rosiglitazone increased PPARγ activity, likely leading to beneficial effects on mitochondrial respiration, coupling efficiency, number, mass, and fusion. We also observed a significant reduction in lipid droplet size, which may result from enhanced mitochondrial oxidative metabolism, not beiging, as we did not detect *UCP1* upregulation after treatment. This aligns with previous findings indicating that the mitochondrial oxidation of fatty acids derived from lipid droplets requires mitochondrial fusion,^42^ suggesting a close relationship between lipid droplet metabolism and mitochondrial dynamics. Improvements in mitochondrial outcomes were accompanied by a restored capacity for adiponectin secretion. This is consistent with existing literature linking mitochondrial function to adiponectin secretion,^43^ and the *ADIPOQ* gene being a known downstream target of PPARγ.^28^ While rosiglitazone is known for its insulin-sensitizing effects, no such effect was seen in our Akt phosphorylation analysis. This indicates that the insulin-sensitizing properties of rosiglitazone may operate independently of enhanced insulin signaling via Akt, as previously suggested,^44^ warranting further investigation of its insulin-sensitizing effects in our adipocyte spheroid model. Overall, our study demonstrates that the first positive outcomes of rosiglitazone treatment include improved mitochondrial metabolism, reduced lipid droplet size, and restored adiponectin secretion. These findings also underscore our adipocyte spheroid model as a valuable tool for studying mitochondrial boosters and, although not investigated in the present study, assessing potential mitochondrial toxicity in drug development.

In conclusion, we have developed a new *in vitro* model of human adipocyte spheroids, which will allow us and others to investigate human white adipocyte mitochondrial biology in physiological and pathophysiological conditions. Furthermore, our model can potentially serve as an *in vitro* drug testing platform in the anti-obesity drug discovery field. A better understanding of the molecular mechanisms behind adipocyte mitochondrial dysfunction, along with the identification of mitochondria activating compounds, could provide tools for mitochondrial pharmacotherapies and the treatment of obesity-related metabolic disorders.

### Limitations of the study

In this study, we mainly used commercially available preadipocytes to increase model and data transparency. However, despite this benefit, the use of these preadipocytes from two middle-aged healthy female donors, one a person with normal weight and the other a person with overweight, was also a limitation. Future studies should include male-derived cells to assess potential sex-related differences. More broadly, additional work is needed to clarify the influence of donor characteristics *e.g.*, BMI, sex, age, and metabolic diseases, such as diabetes, on the differentiation and functionality of adipocyte spheroids. Moreover, as our adipocyte spheroids were generated using only preadipocytes, the absence of other cell types typically present in scWAT likely influenced our results. For example, due to the absence of other cell types, the lipid droplet morphology, metabolic, and endocrine profile of our adipocyte spheroids may not fully recapitulate those of *in vivo* mature white adipocytes.

Another limitation of our model is the use of GFR-Matrigel, the only animal-derived component in our model, which may induce some non-physiological signals. However, as compared to other scaffolds, only GFR-Matrigel has been proven to provide an optimal ECM stiffness and composition for adipogenic differentiation in human 3D adipocyte cultures.^11^ One of the disadvantages of GFR-Matrigel is uneven and poorly controlled incorporation into adipocyte spheroids, which increases experimental variation. To overcome these GFR-Matrigel-related issues, a high number of replicates is recommended for each experiment.

Lastly, current fluorescence live cell imaging techniques do not allow the imaging of the whole adipocyte spheroids throughout their entire height and length. Consequently, we have been able to acquire information related to the differentiation efficiency and lipid droplet and mitochondrial morphology only from the outer layer of the adipocyte spheroids. However, cryosectioning revealed the presence of differentiated adipocytes also in the core of the adipocyte spheroids. In any case, we present live images of human adipocyte spheroids, which are likely to provide more biologically relevant data than images from fixed adipocytes.

## Resource availability

### Lead contact

Further information and requests for resources should be directed to and will be fulfilled by the lead contact, Eija Pirinen (eija.pirinen@oulu.fi).

### Materials availability

This study did not generate new unique reagents.

### Data and code availability

• Data reported in this article will be shared by the lead contact upon request.
• This article does not report original code.
• Any additional information required to reanalyze the data reported in this article is available from the lead contact upon request.

## Acknowledgments

We would like to express our gratitude to Tilkka Hospital, Helsinki, Finland, for their assistance in tissue sample collection and preparation. Imaging was performed at the Biomedicum Imaging Unit, and FIMM High Content Imaging and Analysis unit services of the University of Helsinki, supported by the Helsinki Institute of Life Science (HiLIFE) and 10.13039/501100013840Biocenter Finland. These core facilities were supporting imaging and data analysis. We also thank the Chair of Livestock Biotechnology Unit at TUM for their help in freezing spheroids, and the Chair of Nutrition and Immunology at TUM for their assistance with spheroid processing for imaging and cryosectioning. We extend our thanks to Nick Howe from Agilent Technologies for his invaluable help in establishing the Seahorse protocol for adipocyte spheroids. Additionally, we acknowledge Minna Eriksson and Emma Paasikivi for their technical support, Riikka Jokinen for providing materials, and Juha Hulmi for analytical support. This work was supported by the Academy of Finland/Research Council of Finland (335445, 335446, 314455, 314456, 266286, 272376, 314383, and 335443), 10.13039/100008723Finnish Medical Foundation, 10.13039/501100013500Finnish Diabetes Research Foundation, Suorsa Foundation, Gyllenberg Foundation, 10.13039/501100009708Novo Nordisk Foundation (NNF20OC0060547, NNF17OC0027232, NNF10OC1013354), 10.13039/501100007417Paulo Foundation, 10.13039/501100006306Sigrid Jusélius Foundation, Government Research Funds, Research Council of Finland Profi6 funding (336449) awarded to the University of Oulu. C.E.H. was supported by Karolinska Institutet (2-1062/2018 and 2-189/2022) and Diabetes Wellness Sweden (DWPG-2022-0032). A.W. was awarded a young investigator start-up project funded by the 10.13039/501100001659Deutsche Forschungsgemeinschaft (CRC-TRR333 BATenergy). Open access was funded by Helsinki University Library.

## Author contributions

A.W., A.I., C.E.H., and E.P. conceptualized the model. A.W., J.H.L-K., K.P., A.H., M.K., L.R., A.I., S.M., S.S., S.L., M.T.R., S.K., A.O., and E.P. performed experiments and/or analyzed data. A.W., J.H.L-K., K.P., and E.P. wrote the article. A.W., J.H.L-K., A.H., and I.R. prepared the figures and tables. K.H., E.H., P.E., O.U., M.M., H.P., K.H.P., M.K., K.A.V., C.E.H., and E.P. provided resources, all co-authors critically reviewed, and edited the article. All authors approved the final version of the article.

## Declaration of interests

The authors declare no competing interests.

## Declaration of generative AI and AI-assisted technologies in the writing process

During the preparation of this work, the authors used ChatGPT and Copilot to improve readability and check grammar. After using these tools, the authors reviewed and edited the content as needed and take full responsibility for the content of the published article.

## STAR★Methods

### Key resources table

**Table:** 

REAGENT or RESOURCE	SOURCE	IDENTIFIER
**Antibodies**		

p-Akt^Ser473^	Cell signaling	9271; RRID: AB_329825
Akt	Cell signaling	9272; RRID: AB_329827
Vinculin	Abcam	ab129002; RRID: AB_11144129

**Biological samples**		

Human preadipocytes BMI 23 (Donor 1)	Lonza	SAT-PT-5020, TAN 33226
Human preadipocytes BMI 28 (Donor 2)	Lonza	PT5020, TAN 27458
ScWAT biopsies collected from the abdominal area (Donors 3 to 9)	Tilkka Hospital, Helsinki	N/A
Subcutaneous abdominal MAFs (Donors 3 to 9)	Tilkka Hospital, Helsinki	N/A

**Chemicals, peptides, and recombinant proteins**		

3-Isobutyl-1-Methylxanthin	Sigma-Aldrich	I7018
Accumax	StemmCell Technoligies	7921
Adipocyte Differentiation Medium	PELOBiotech	PB-CDH-442-3699
Adipocyte Maintenance Medium	PELOBiotech	PB-C-MH-442-3699-M
AdipoRed™ Assay Reagent	Lonza	PT-7009
Amphotericin B	Sigma-Aldrich	A2924
Amplex Ultrared Reagent	Thermo Fisher Scientific	A36006
Aquatex	Merck	108562
Biotin	Sigma-Aldrich	B4639
Bodipy™ 493/503	Thermo Fisher Scientific	D3922
Chemically Defined Lipid Concentrate	Thermo Fisher Scientific	11905031
Dexamethasone	Sigma-Aldrich	D1756
DMEM	Gibco	11880
DMEM/F12 (1:1)	Gibco	21331
Eosin	Histolab	1650
Free Glycerol Reagent	Sigma-Aldrich	F6428
Gentamicin	Sigma-Aldrich	G1397
Glutamax	Gibco	35050-038
GFR-Matrigel	Corning	356231
Haematoxylin	Histolab	01820
Hoechst 33342	Sigma-Aldrich	H3570
Human Insulin (for 3D cultures)	Sigma-Aldrich	I9278
Human Insulin (for 2D cultures)	Sigma-Aldrich	I5500
Human Serum	Biowest	S4190
Isoproterenol Hydrochloride	Sigma-Aldrich	I6504
MitoTracker Red CMXRos	Thermo Fisher Scientific	M7512
Panthotenate	Sigma-Aldrich	P5155
Penicillin-Streptomycin	Gibco	15070-063
Protease and Phosphatase Inhibitor Cocktail	Thermo Fisher Scientific	78442
Poly-D-lysine	Sigma-Aldrich	P6407
Proteinase K	Macherey-Nagel	740506
RNase A	Thermo Fisher Scientific	EN0531
Rosiglitazone	Calbiochem	557366
Roti®-histokitt	Carl Roth	6638.2
Seahorse XF Base Medium	Agilent Technologies	103335-100
Seahorse XF Calibrant Solution	Agilent Technologies	100840-000
SYBR Green Master Mix	Thermo Fisher Scientific	K0252
Tissue Tek O.C.T. Compound	Histolab	45830
Trizol^TM^ Reagent	Invitrogen	15596018

**Critical commercial assays**		

12-230 kDa Separation Module	ProteinSimple, Bio-Teche	SM-W004
Anti-Rabbit Detection Module	ProteinSimple, Bio-Teche	DM-001
CyQuant™ Cell Proliferation Assay	Thermo Fisher Scientific	C7026
Ez-PCR Mycoplasma Detection Kit	Biological industries	20-700-20
Human Leptin ELISA Kit	Ray Biotech	ELH-Leptin
Human MCP-1 ELISA Kit	Abcam	Ab179886
Human Total Adiponectin/Acrp30 Immunoassay	RnD Systems	DRP300
LDH Kit	Thermo Fisher Scientific	981906
RNeasy Micro Kit; RNeasy Mini Kit	Qiagen	74004; 74104
Seahorse XFe Cell Mito Stress Test Kit	Agilent Technologies	103015-100
Superscript™ VILO™ cDNA Synthesis Kit	Invitrogen	10499763

**Oligonucleotides**		

*ADIPOQ* 5′-TGGTGAGAAGGGTGAGAA-3′ 5′-AGATCTTGGTAAAGCGAATG-3′	Metabion International AG	N/A
*CD36* 5′-CAGGTCAACCTATTGGTCAAGCC-3′ 5′-GCCTTCTCATCACCAATGGTCC-3′	Metabion International AG	N/A
*FASN* 5′-CCGAGACACTCGTGGGCTA-3′ 5′-CTTCAGCAGGACATTGATGCC-3′	Metabion International AG	N/A
*HSL* 5′-AGCCTTCTGGAACATCACCGAG-3′ 5′-TCGGCAGTCAGTGGCATCTCAA-3′	Metabion International AG	N/A
*IPO8* 5′-AAGAAACCGCGCTTGAGGGG-3′ 5′-ATCCTCGCTGAGTGGTGCCA-3′	Metabion International AG	N/A
*LEPTIN* 5′-GCTGTGCCCATCCAAAAAGTCC-3′ 5′-CCCAGGAATGAAGTCCAAACCG-3′	Metabion International AG	N/A
*LXRα* 5′-TGGACACCTACATGCGTCGCAA-3′ 5′-CAAGGATGTGGCATGAGCCTGT-3′	Metabion International AG	N/A
*PPARγ* 5′-TACTGTCGGTTTCAGAAATGCC-3′ 5′-GTCAGCGGACTCTGGATTCAG-3′	Metabion International AG	N/A
*UCP1* 5′-CAAATCAGCTCCGCCTCTCT-3′ 5′-AATGAATACTGCCACTCCTCCAG-3′	Metabion International AG	N/A
*16S* (mtDNA) 5′-GGGGCGACCTCGGAGCAGAA-3′ 5′-ATAGCGGCTGCACCATCGGGA-3′	Metabion International AG	N/A
*CYTB* (mtDNA) 5′-GCCTGCCTGATCCTCCAAAT-3′ 5′-AAGGTAGCGGATGATTCAGCC-3′	Metabion International AG	N/A
*DLOOP* (mtDNA) 5′-CATCTGGTTCCTACTTCAGGG-3′ 5′-CCGTGAGTGGTTAATAGGGTG-3′	Metabion International AG	N/A
*APP* (genomic DNA) 5′-TGTGTGCTCTCCCAGGTCTA-3′ 5′-CAGTTCTGGATGGTCACTGG-3′	Metabion International AG	N/A
*B2M* (genomic DNA) 5′-TGCTGTCTCCATGTTTGATGTATCT-3′ 5′-TCTCTGCTCCCCACCTCTAAGT-3′	Metabion International AG	N/A
*HBB* (genomic DNA) 5′-CAGGTACGGCTGTCATCAGTTAG-3′ 5′-CATGGTGTCTGTTTGAGGTTGCT-3′	Metabion International AG	N/A

**Software and algorithms**		

CFX Maestro Software	Bio-Rad	N/A
Compass for Simple Western software version 6.2.0(ProteinSimple)	Bio-Techne	N/A
Fiji/ImageJ software	ImageJ	https://imagej.nih.gov/ij/
GraphPad Prism software version 10.1.2.	GraphPad Software	N/A
Harmony 4.9 software	Revvity, Inc.	https://www.revvity.com/de-en/product/harmony-5-2-office-revvity-hh17000019
LightCycler 480 SW 1.5	Roche Diagnostic AG	N/A
qBase+ software version 3.4 (Biogazelle)	Biogazelle	N/A
ViewPoint Light	PreciPoint GmbH	N/A

**Other**		

Ultra-low attachment cell culture plates	Greiner	650970
iSpacers, 0.2 mm	SunJin Lab	IS007
OptiPlate 96-well plates	PerkinElmer	6005270
Seahorse XFe 96 Spheroid Microplates	Agilent Technologies	102978-100

### Experimental model and study participant details

#### Cell sources and isolation methods

##### Commercial cells

Human subcutaneous preadipocytes were obtained from two healthy female White donors without diabetes or other metabolic diseases (Lonza, Basel, Switzerland). Donor 1 had a BMI of 23 (Cat# SAT-PT-5020; TAN 33226), and donor 2 had a BMI of 28 (Cat# PT-5020; TAN 27458) (Table 1). Cells from both donors were confirmed to be free of mycoplasma contamination using the EZ-PCR Mycoplasma Detection Kit (Cat# 20-700-20, Biological Industries) according to the manufacturer’s instructions. Briefly, media were collected 24 h after the last medium change and stored in -20°C until use. After thawing, media were centrifuged at 250 x g briefly to remove cellular debris. Supernatants were transferred into a new tube followed by a centrifugation for 10 minutes (min) at 16,000 x g at room temperature (RT). Pellets were resuspended into 50 μL of buffer solution supplied by the manufacturer, followed by a 3 min denaturation at 95°C. PCR amplification was conducted by mixing the sample, reaction mix, internal control DNA template, internal control primers mix and water. Positive and negative controls were included. Next, samples were loaded into a thermal cycler, and the following protocol was used: 94°C for 30 seconds (sec), then 35 cycles of 94°C for 30 sec, 60°C for 120 sec and 72°C for 60 sec. Amplified products mixed with a loading buffer were loaded into 2% agarose gel for gel electrophoresis. Gel was imaged (BioRad Laboratories) and samples were verified negative for mycoplasma contamination by comparison to positive and negative controls.

##### Mature adipocytes and tissue biopsies

Human MAFs and scWAT biopsies were collected from the abdominal region of seven healthy female White donors undergoing an elective liposuction at Tilkka Hospital, Helsinki (donors 3–9; see Table 1). Donors ranged from 36 to 53 years of age, with BMI values between 19–30. Immediately post-surgery, scWAT samples were dissected to remove connective tissue and vasculature. Tissue was split for downstream applications into two parts, whereas one part was snap-frozen in liquid nitrogen and stored at –80°C.

##### MAF isolation

One part of each scWAT sample was used for MAF isolation via mechanical and enzymatic digestion, as previously described.^21^ Briefly, tissue samples were minced into small fragments and digested in DMEM/F12 medium (Cat# 21331-020, Thermo Fisher Scientific) containing collagenase type I (1.5 mg/mL; Cat# 17100017, Thermo Fisher Scientific) for 60–90 min with gentle shaking at RT. The resulting cell suspension was centrifuged for 10 min at 600 × *g* at RT to separate the MAF from the SVF. The MAF layer was collected and stored at −80°C.

##### Ethics statement

All tissue donors provided written informed consent. The study protocol was reviewed and approved by the Ethics Committee of Helsinki University Hospital (Approval ID: HUS/1039/2019).

### Method details

#### Cell culture

##### Preadipocyte expansion for spheroid formation

Human subcutaneous preadipocytes were cultured in growth medium containing DMEM (3.15 g/L glucose; Cat# 21331, Thermo Fisher Scientific) supplemented with 5% human serum (Cat# S4190-100, Biowest), 1% GlutaMAX (Cat# 35050, Thermo Fisher Scientific), 15 ng/mL amphotericin B (Cat# A2924, Sigma-Aldrich), and 30 μg/mL gentamicin (Cat# G1397, Sigma-Aldrich). Cells were maintained at 37°C in a humidified incubator with 5% CO_2_ and typically expanded for 4–6 passages before use in experiments. To ensure similar passaging and cellular senescence, the splitting ratio was 1:4 throughout the study. For cell detachment, 0.25% trypsin (Cat# 15090046, Thermo Fisher Scientific) was used for 5 min at 37°C. Serum-containing growth medium was used to inactivate trypsin.

##### Adipocyte spheroid formation

Preadipocytes were seeded into 96-well round-bottom cell-repellent plates (Cat# 650970, Greiner Bio-One) at 15,000, 25,000, or 35,000 cells/well in 200 μL of serum-free growth medium. After a 48-hour spontaneous aggregation, undifferentiated spheroids were used for the experiments as preadipocyte spheroids.

##### Differentiation protocols for adipocyte spheroids

After a 24-hour spontaneous aggregation, 150 μL of growth medium was removed and 30 μL of GFR-Matrigel (Cat# 356231, Corning) was added per well followed by gentle pipetting up and down approximately 5 times to enhance GFR-Matrigel incorporation into the adipocyte spheroid. Next, after a 30-min incubation of the plate at 37°C to polymerize GFR-Matrigel, 150 μL of adipocyte differentiation medium (DM; 1 g/L glucose) without serum and thiazolidinediones was added per well. DM was a whole component kit composed of 1 μM dexamethasone, 1 μg/mL human insulin, 500 μM 3-isobutyl-1-methylxanthine, 100 μM indomethacin, and additional proprietary components from the PELOBiotech kit (Cat# PB-CDH-442-3699). Differentiation was continued for either 7 days (2-week protocol), 14 days (3-week protocol) or 20 days (4-week protocol). DM was refreshed every 2-3 days by removing 150 μL of medium well by well with FinnTip Wide Orifice tips (Cat# 9405123, Thermo Fisher Scientific) and by adding 150 μL of fresh medium per well with a multichannel pipette.

After differentiation, DM was replaced with adipocyte maintenance medium (MM; Cat# PB-C-MH-442-3699-M, PELOBiotech) containing 1 g/L glucose, 1 μg/mL human insulin and additional proprietary components from the kit. Maintenance phase lasted for 7 days for all the differentiation protocols. MM was refreshed every 2-3 days similarly as described for DM. Notably, for both DM and MM, kit containing antibiotics were replaced by amphotericin B and gentamicin as described for growth medium.

##### LM exposure in adipocyte spheroid cultures

To mimic nutrient-based energy surplus-induced fat accumulation and metabolic complications, adipocyte spheroids from the donor 2 with BMI 28 were administered with Chemically Defined Lipid Concentrate (Cat# 11905031, Thermo Fisher Scientific) in 1:125 ratio with medium throughout the full 3-week protocol, including both adipocyte differentiation and maintenance phases.

##### Pharmacological treatment for adipocyte spheroids

To evaluate drug responsiveness, adipocyte spheroids were first cultured with or without 1:125 LM throughout the standard 3-week protocol. Next, LM-free spheroids were treated for 72 h with vehicle (0.1% DMSO), while LM-administered spheroids received either vehicle or 1 μM rosiglitazone (Cat# 557366, Calbiochem) in 0.1% DMSO. Treatments were conducted in MM.

##### Monolayer adipocyte cultures and differentiation for 2D versus 3D comparison

Monolayer adipocyte differentiation was performed as previously described.^21^ Briefly, preadipocytes from the donors 1 and 2 were seeded and cultured until reaching confluence using DMEM/F12 (Cat# 21331, Thermo Fisher Scientific) growth medium supplemented with 5% human serum (Cat# S4190-100, Biowest), 1% GlutaMAX (Cat# 35050, Thermo Fisher Scientific) and 1% penicillin-streptomycin (Cat# 15070-063, Thermo Fisher Scientific). Next, growth medium was completely removed and replaced with differentiation medium, which was the same as growth medium with the following additional supplements; 33 μM pantothenate (Cat# P5155, Sigma-Aldrich), 17 μM biotin (Cat# B4639, Sigma-Aldrich-Aldrich), 100 nM human insulin (Cat# I5500, Sigma-Aldrich), 1 μM dexamethasone (Cat# D1756, Sigma-Aldrich), 1 μM rosiglitazone (Cat# 557366, Calbiochem) and 500 μM 1-methyl-3-Isobutylxanthine (Cat# I7018, Sigma-Aldrich). The cells were maintained in differentiation medium for 7 days during which medium was refreshed twice.

Next, differentiation medium was replaced with maintenance medium. Maintenance medium composition was the same as for differentiation medium with the exception of excluding rosiglitazone and 1-methyl-3-isobutylxanthine. Maintenance medium was refreshed every 4–5 days for a total of 14 days.

#### RNA extraction, cDNA synthesis and real-time quantitative PCR (RT-qPCR)

##### RNA extraction

Total RNA was extracted from 3D cultured adipocyte spheroids and 2D differentiated adipocytes derived from the donors 1 and 2 as well as from scWAT and MAFs from the donors 3-9 (Table 1). For 3D samples, 12-15 adipocyte spheroids were pooled per sample, while the cells from one well from a 6-well plate were used per sample. All samples were processed using TRIzol™ Reagent (Cat# 15596018, Invitrogen). Adipocyte spheroids were homogenized using the Kimble® Pellet Pestle® Cordless Motor with matching sterile grinders (DWK Life Sciences GmbH), while 2D differentiated adipocytes, scWAT and MAFs were homogenized using TissueLyser LT (4 min, 45 Hz, Qiagen) and a metal bead. All homogenates were centrifuged for 10 min at 12,000 × g at 4°C. Chloroform (20% of the initial sample volume) was added to each supernatant, followed by 3 min incubation at RT. Samples were centrifuged again for 30 min at 12,000 × g at 4°C. The aqueous top phase was transferred to a new tube and mixed with 80% ethanol in 1:1 ratio. RNA was purified according to the manufacturer’s protocol using the RNeasy Micro Kit (Cat# 74004, Qiagen) for adipocyte spheroids and 2D differentiated adipocytes and the RNeasy Mini Kit (Cat# 74104, Qiagen) for scWAT and MAFs samples with the except of omitting the DNase treatment. Briefly, samples were loaded into spin columns, centrifuged for 1 min at 10,000 × g at RT, washed with 700 μL RW1 followed by two washings with 500 μL RPE buffer (first wash: 1 min at 10,000 × g; second: 2 min at full speed). RNA was eluted with 10–14 μL RNase-free water and stored at -80°C.

##### scDNA synthesis

Reverse transcription was performed using the SuperScript™ VILO™ cDNA Synthesis Kit (Cat# 10499763; Invitrogen) following the manufacturer’s protocol. Varying RNA amount (700–1000 ng) was used for all sample types, combined with 4 μL 5× VILO Reaction Mix and 2 μL 10× SuperScript Enzyme Mix provided in the kit, and RNase-free water was added to a final volume of 20 μL per reaction. Reactions were run at 25°C for 10 min, 42°C for 120 min, and 85°C for 5 min, followed by a hold at 4°C until samples were stored at -20°C.

##### RT-qPCR

RT-qPCR was performed using SYBR Green Master Mix (Cat# K0252, Thermo Fisher Scientific) with 15 ng of cDNA template per well, independent of sample origin. A 96-well plate with a total reaction volume of 20 μL/well was used for the RT-qPCR analysis. A master mix was prepared by mixing 10 μL Maxima SYBR Green/ROX qPCR Master Mix (2x), 2 μL primer mix and 6 μL nuclease-free water. PCR amplification was carried out on a CFX96 Real-Time PCR Detection System (Bio-Rad Laboratories) with an initial denaturation at 95°C for 3 min, followed by 39 cycles of denaturation at 95°C for 10 sec, annealing at 62°C for 30 sec, and extension at 72°C for 60 sec. Fluorescence detection occurred after each full cycle. A melt curve analysis was performed from 65°C to 95°C in 0.5°C increments, holding for 5 sec at each step. The data were analyzed using CFX Maestro software.

##### Data analysis

Gene expression was analyzed using the efficiency-corrected ΔΔCt method. Of the tested housekeeping genes, *IPO8* (importin 8) was selected as the internal control due to its stable expression and minimal variance across sample groups. Primer sequences are listed in Key resource table.

#### Fluorescence live cell imaging

##### Live-cell staining of adipocyte spheroids

(Pre)adipocyte spheroids were washed twice with 1× DPBS (Cat# 14190094, Thermo Fisher Scientific) and incubated for 20 min in the dark on a shaker (30 rpm) at RT in staining solution containing 0.5 μg/mL BODIPY 493/503 (Cat# D3922, Thermo Fisher Scientific) to label lipid droplets, 1 μg/mL Hoechst 33342 (Cat# H3570, Sigma-Aldrich) to label nuclei and 100 nM MitoTracker Red CMXRos (Cat# M7512, Thermo Fisher Scientific) to label mitochondria. The staining solution was prepared in 1× DPBS.

##### Mounting for confocal imaging

Following staining, (pre)adipocyte spheroids were washed twice in 1× DPBS for 5 min. Four iSpacers (0.2 mm, Cat# IS007, SunJin Lab, Taiwan) were placed on the corners of a glass slide. 100 μL of Aquatex® mounting medium (Cat# 108562, Merck) was added to the center. (Pre)adipocyte spheroids were transferred into Aquatex®, covered with a coverslip, sealed using non-fluorescent transparent nail polish, and stored at 4°C until imaging.

##### Confocal imaging

Images were acquired using a PerkinElmer Opera Phenix® High-Content Screening system with either a 40× air objective (NA 0.6) or a 40× water immersion objective (NA 1.1). The following excitation channels were used: 350 nm (Hoechst), 488 nm (BODIPY) and 568 nm (MitoTracker).

##### Image analysis and quantification

Fluorescence image analysis was performed using Harmony 4.9 software (PerkinElmer). Analysis pipelines are described in detail in Table S2. The following parameters were quantified per spheroid: lipid droplets: diameter, area, and number; mitochondria: number, mass, and morphology. Differentiation efficiency was calculated as the percentage of lipid-containing cells relative to total nuclei count (non-lipid-bearing cells were not excluded beforehand).

For representative live-cell fluorescent images of nuclei, lipid droplets and mitochondria shown in Figures 1 and 2, Figures 3 and 4 and Figures 5 and 6, each pair of figures was acquired from the same zoomed-in regions. This approach ensured comparability, spatial correspondence and scalability between nuclei, lipid droplet and mitochondria structures. The separate fluorescence channels were subsequently combined to generate merged images, which are presented in Figure S4, allowing visualization of the relative positions and sizes of nuclei, lipid droplets, and mitochondria within the same cellular region.

##### Image processing and dataset filtering

Background correction was applied to enhance lipid droplet quantification, with particular emphasis on LM-administered spheroids. To eliminate false-positive lipid-bearing cells in undifferentiated preadipocyte spheroids, donor-specific thresholds were established using the average mean lipid droplet length (μm) from undifferentiated controls: 2.27 μm for donor 1 and 0 μm for donor 2. Only cells exceeding these thresholds were included in lipid droplet analysis for differentiated adipocyte spheroids. For mitochondrial analysis, cells lacking any detectable MitoTracker signal were excluded before quantification.

#### Mitochondrial respiration

##### Plate preparation and calibration

One day prior to the run, the Seahorse XFe96 Analyzer (Agilent Technologies) was calibrated by incubating the Agilent Seahorse XFe96/XF Pro sensor cartridge in Seahorse XF Calibrant (Cat# 100840-000, Agilent Technologies) overnight at 37°C in a humidified, non-CO_2_ incubator.

On the assay day, (pre)adipocyte spheroids were washed with 1× DPBS and transferred into Seahorse XFe96 Spheroid Microplate (Cat# 102978-100, Agilent Technologies). To promote attachement, microplate was pre-coated with 30 μL of 0.1 mg/mL poly-D-lysine (Cat# P6407, Sigma-Aldrich) for 30 min followed by washing with distilled water.

##### Assay medium and conditions

The assay medium consisted of Seahorse XF Base medium (Cat# 103335-100, Agilent Technologies) supplemented with 10 mM glucose (Cat# 10141520, Thermo Fisher Scientific), 2 mM GlutaMAX (Cat# 35050, Thermo Fisher Scientific), and 1 mM sodium pyruvate (Cat# S8636, Sigma-Aldrich). Where applicable, LM, rosiglitazone, or DMSO were added at experimental concentrations consistent with the prior treatments. The medium pH was confirmed to be 7.4 before loading the (pre)adipocyte spheroids.

##### Injection protocol and compounds

(Pre)adipocyte spheroid mitochondrial function was evaluated using the Seahorse XF Cell Mito Stress Test Kit (Cat# 103015-100; Agilent Technologies) according to the manufacturer’s instructions. Briefly, compounds were prepared in assay medium to stock concentrations of 100 μM for oligomycin and FCCP, and 50 μM for rotenone and antimycin A. After the drugs were delivered via injection ports, the final concentrations were as follows: oligomycin (2.5 μM; ATP synthase inhibitor), FCCP (1.5 μM; mitochondrial uncoupler), and rotenone plus antimycin A (1 μM each; complex I and III inhibitors, respectively). The measurement protocol consisted of the following cycle ranges: baseline: 4–8 cycles; oligomycin: 10–18 cycles; FCCP: 12–18 cycles; rotenone/antimycin A: 12–25 cycles.

Each cycle consisted of 2 min mixing, 2 min equilibration, and 2 min measurement steps. The cycle number for each condition was optimized per run to ensure a stable OCR plateau.

##### Post-assay processing and normalization

Following the run, medium was removed, spheroids were washed with 1× DPBS and the plate was stored at –80°C for later DNA quantification (see section “total DNA content”) used for the normalization of the OCR data.

##### Data processing and calculation of mitochondrial parameters

The OCR data were analyzed using Seahorse Wave Desktop Software (Agilent) and processed in Microsoft Excel. The following mitochondrial parameters were calculated: basal respiration, ATP-linked respiration, proton leak, maximal respiration, spare respiratory capacity, and coupling efficiency.

Basal and maximal respiration were determined by selecting the three highest consecutive OCR values before and after FCCP injection, respectively. The two to three lowest OCR values following rotenone/antimycin A injection were used to determine non-mitochondrial respiration for baseline correction. The final OCR values were normalized to total DNA content per well.

#### Total DNA content

##### DNA extraction and lysis

(Pre)adipocyte spheroids were homogenized in 30 μL of self-made lysis buffer containing 0.5 M NaOH (Cat# 6771.1, Carl Roth), 180 mM NaCl (Cat# 3957.1, Carl Roth), and 1 mM EDTA (Cat# 8043.2, Carl Roth). Samples were incubated at 37°C for 1–2 h to ensure complete lysis.

##### Total DNA quantification

Total DNA content was quantified using the CyQUANT™ GR dye (Cat# C7026, Thermo Fisher Scientific) in combination with a custom GR dye buffer prepared by mixing self-made lysis buffer (see section “DNA extraction and lysis”) with 1 M Tris–HCl at a 1:10 ratio. The GR dye stock was diluted 1:200 into this buffer to prepare a final working solution.

First, 10 μL of the (pre)adipocyte spheroid lysate was transferred to a black 96-well OptiPlate (Cat# 6005270 PerkinElmer) and 90 μL of 1 M Tris-HCl (Cat# 9090.2, Carl Roth) was added per well to neutralize the lysate. Next, RNase A (Cat# EN0531, Thermo Fisher Scientific) was added to a final concentration of 100 μg/mL, and samples were incubated for 30 min at 37°C. Finally, 100 μL of the 1:200 diluted GR dye was added per well. Fluorescence detection was performed on two systems depending on the application: mitochondrial respiration experiments were read using a Tecan Infinite M200 plate reader (Tecan Trading AG, Switzerland) with 480/520 nm excitation/emission settings, while lipolysis experiments were analyzed on a VICTOR3 V 1420 Multilabel Counter (PerkinElmer) at 485/535 nm.

Note that raw fluorescence values were adjusted for the lysate dilution factor (1:3). In contrast to the manufacturer's protocol, the supplied standards were not used and 1:9 mixture of the self-made lysis buffer and 1 M Tris–HCl was used as the diluent for dye preparation.

#### Bright-field microscopy

##### Bright-field imaging

Undifferentiated preadipocyte spheroids (day 2) and differentiated adipocyte spheroids (day 21 or 24) were imaged directly from U-bottom 96-well plates using a Leica MC120 HD microscope (Leica Microsystems GmbH) at 4× magnification. Images were captured using a Lilliput HD camera and 7″ monitor system (Zhangzhou, China).

##### Adipocyte spheroid diameter measurement

The diameter of the (pre)adipocyte spheroids was analyzed using Fiji/ImageJ software (version 1.54f; National Institutes of Health) on a Windows platform.

##### Cell viability

Cell viability of adipocyte spheroids was assessed by quantifying LDH release into the culture medium using the LDH kit (Cat# 981906, Thermo Fisher Scientific). Media samples were collected after treatment (see section “cell culture”) and analyzed on an automated Indiko Plus Analyzer (Thermo Fisher Scientific) following the manufacturer’s protocol (Cat# 98640000, Thermo Fisher Scientific). Briefly, 6 μL of medium was mixed with 100 μL of LDH_IFCC A reagent and samples were incubated for 400 sec at 37°C. Next, 25 μL of LDH_IFCC B reagent was added after 90 sec of incubation time at 37°C, absorbance was measured at 340 nm. Absorbance was measured every 18 sec for 180 sec to enable analysis of kinetic enzyme activity.

#### Intracellular triglyceride content

##### Adipocte spheroid dissociation

Individual (pre)adipocyte spheroids were dissociated using Accumax (Cat# 07921, StemCell Technologies). Spheroids were first washed with 1× DPBS transferred into a black OptiPlate 96-well plate in 30 μL volume and incubated with 70 μL of Accumax per well. The plate was placed on a thermomixer (PHMP, Grant Instruments Ltd, UK) for 40 min at 37°C and 1200 rpm. Dissociation was enhanced by pipetting each well 10 times at 10-min intervals (4 total pipetting steps).^45^ Following dissociation, cells were used directly for triglyceride quantification.

##### Triglyceride quantification

Intracellular triglyceride content was assessed using AdipoRed™ Assay Reagent (Cat# PT-7009, Lonza) according to the manufacturer’s instructions. Briefly, after adding 2.5 μL of AdipoRed™ Assay Reagent per well followed by a 10 min incubation, fluorescence was measured at excitation/emission wavelengths of 485/570 nm using a VICTOR2 Wallac 1420 Multilabel Counter (PerkinElmer). To normalize for cell number, nuclei were stained with 1 μg/mL Hoechst 33342 (Cat# H3570, Thermo Fisher Scientific), and fluorescence was recorded at 355/460 nm. Triglyceride levels were expressed as AdipoRed fluorescence intensity normalized to Hoechst signal per well.

#### Adiponectin, MCP-1, and leptin ELISAs

##### Sample collection for adipocyte spheroids and monolayer cultures

Conditioned media were collected from differentiated adipocyte spheroids and monolayer cultures on day 21 of differentiation, 3 days after the final medium change. For undifferentiated preadipocyte spheroids and 2D preadipocytes, media were collected after 2 days of culture in standard growth medium. All media samples were stored at –80°C.

##### Adiponectin ELISA analysis

Adiponectin concentrations in culture medium were measured using the Human Adiponectin/Acrp30 Quantikine ELISA Kit (Cat# DRP300; R&D Systems) according to the manufacturer’s instructions. Briefly, standards and 1:2 (2D differentiated adipocytes) or undiluted samples (all the other groups) were added to wells pre-coated with a monoclonal antibody specific for human adiponectin and incubated for 2 h at RT. After washing, wells were incubated with adiponectin–conjugated detection antibody for 2 h at RT. Following additional washes, substrate solution was added and incubated for 20 min at RT. The reaction was stopped with the stop solution, and absorbance was measured at 450 nm with wavelength correction at 550 nm. The mean minimum detectable dose of the assay was 0.246 ng/mL.

##### MCP-1 ELISA analysis

Monocyte chemoattractant protein-1 (MCP-1/CCL2) concentrations in culture medium were measured using the Human MCP-1 ELISA Kit (Cat# ab179886; Abcam) according to the manufacturer’s protocol. Briefly, standards and 1:5 diluted samples were added with the capture and detector antibody cocktail and incubated for 1 h at RT. After washing, samples were incubated for 10 min with TMB development solution, stopped with stop solution, and absorbance was measured at 450 nm. The sensitivity of the assay was 1.26 pg/mL.

##### Leptin ELISA analysis

Leptin concentrations in culture supernatants were measured using a Human Leptin ELISA Kit (Cat# ELH-Leptin, RayBiotech) following the manufacturer’s protocol. Briefly, standards as well as 1:2 diluted (2D differentiated groups) and undiluted (all the other groups) samples were added to antibody-precoated wells and incubated for 2.5 h. After washing, wells were sequentially incubated for 1 h with biotin-conjugated detection antibody, washed and incubated for 30 min with HRP-streptavidin. After washing, wells were incubated for 30 min with TMB one-step substrate, stopped with stop solution, and absorbance was measured immediately at 450 nm. All incubation steps were conducted at RT with gentle shaking. The sensitivity of the assay was 2 pg/mL.

#### Protein extraction and capillary Western blot

##### Insulin stimulation of adipocyte spheroids

(Pre)adipocyte spheroids were cultured in insulin-free DMEM (1 g/L glucose; Cat# 11880, Thermo Fisher Scientific) supplemented with 1% GlutaMAX for 24 h on day 21 of differentiation. On day 22, spheroids were administered for 30 min with either 1× DPBS (vehicle control) or 100 nM human insulin (Cat# I9278, Sigma-Aldrich) in fresh DMEM with 1% GlutaMAX. Where indicated, rosiglitazone and/or LM were included in the medium at the same concentrations used as described in the section cell culture. After incubation, adipocyte spheroids were washed with 1× DPBS and stored at –80°C until protein extraction.

##### Protein extraction

Proteins were extracted by adding 50 μL of RIPA buffer (50 mM Tris-HCl, pH 7.6; 150 mM NaCl (71380-1KG-D, Sigma-Aldrich); 1 mM EDTA (Cat# 10264960, Fisher Scientific); 1% Triton X-100 (Cat# X100-100 mL, Sigma-Aldrich); 0.5% deoxycholic acid (Cat#, D6750-25G, Sigma-Aldrich); 0.1% SDS (Cat# 05030-500 mL-F, Sigma-Aldrich) supplemented with 3% Halt Protease and Phosphatase Inhibitor Cocktail (Cat# 78442, Thermo Fisher Scientific) to each spheroid. Spheroids were homogenized using a Bel-Art ProCulture Cordless Homogenizer (Cat# 17455799, Thermo Fisher Scientific) with 2 × 10 sec pulses. Lysates were incubated on ice for 20 min, followed by centrifugation for 20 min at 16,000 × g at 4°C. Supernatants were mixed with fluorescent 5X master mix (Cat# PS-ST01EZ, Protein Simple) in a 4:1 ratio, denatured at 95°C for 5 min, and stored at 4°C for up to 2 weeks prior to analysis.

##### Capillary Western Blot

Capillary Western blot was performed using the JESS system (ProteinSimple, Bio-Techne) with 12–230 kDa separation module (Cat# SM-W004, ProteinSimple) and chemiluminescent detection with anti-rabbit detection module (Cat# DM-001, ProteinSimple) following the manufacturer’s instructions as previously described.^46^ Briefly, after loading biotinylated ladder, 3 μL per well of prepared protein samples, blocking reagent (antibody diluent 2), primary antibody, streptavidin-HRP, horseradish peroxidase-conjugated ready-to-use secondary antibody (Cat# DM-001, ProteinSimple), chemiluminescence substrate (luminol-peroxide mix) and wash buffer to the manufacturer-provided microplate according to the volumes and plate map provided by the manufacturer, the plate was centrifuged for 5 min at 1000 x g at RT. Next, the plate was loaded onto the JESS instrument for chemiluminescence analysis. Note that protein concentration was not quantified prior to loading due to the interference from residual GFR-Matrigel, which contains extracellular matrix proteins that co-extract with adipocyte spheroid lysates and artificially inflate protein measurements. Therefore, equal lysate volumes were loaded per capillary for undifferentiated and differentiated spheroid samples.

##### Antibodies and analysis

Primary antibodies used were:

Phospho-Akt^Ser473^(Cat# 9271, 1:10, Cell Signaling Technology). Total Akt (Cat# 9272, 1:50, Cell Signaling Technology). Vinculin (Cat# ab129002, 1:250, Abcam), used as the loading control. Signal quantification and normalization to Vinculin were performed using Compass for Simple Western software, version 6.2.0 (ProteinSimple, Bio-Techne).

#### Lipolysis

##### Lipolysis assay

(Pre)adipocyte spheroid lipolysis rate was assessed by measuring glycerol release to the culture medium under basal and isoproterenol-stimulated conditions. Spheroids were washed with 1× DPBS and incubated with water (vehicle) or 10 μM isoproterenol (isoproterenol hydrochloride; Cat# I6504, Sigma-Aldrich) for 3 h at 37°C and 5% CO_2_ in 30 μL of Krebs–Ringer lipolysis buffer (136 mM NaCl (Cat# 0278.1000, J.T.Baker), 1 mM NaH_2_PO_4_ (Cat# 1.06346, Merck), 1 mM CaCl_2_ (Cat# C/1500/60, FF-Chemicals), 4.7 mM KCl (Cat# 0509, J.T.Baker), 1 mM MgSO_4_ (Cat# 31420, Riedel-de Haen), 2 mM glucose (Cat# G/0500/53, Fisher), 25 mM HEPES (Cat# H7006, Merck), and 2% fatty acid-free BSA (Cat# A8806, Merck); pH 7.4. After incubation, media were collected, preadipocyte spheroids were washed with 1x DPBS, and all samples were stored at -80°C.

##### Glycerol detection

Media glycerol levels were quantified using Free Glycerol Reagent (Cat# F6428, Sigma-Aldrich) combined with Amplex UltraRed Reagent (Cat# A36006, Thermo Fisher Scientific) at a 1:100 dilution ratio. The reaction was incubated for 15 min at RT, and fluorescence was measured at excitation/emission wavelengths of 550/581 nm using a VICTOR2 Wallac 1420 Multilabel Counter (PerkinElmer).

##### Normalization

Media glycerol concentrations were normalized to total DNA content per well, as described in the “total DNA quantification” section.

#### Mitochondrial DNA content

##### Sample preparation and lysis

For each sample, 13–15 (pre)adipocyte spheroids were pooled. To compare undifferentiated and differentiated spheroids, pooled samples were homogenized in 500 μL ice-cold 1× DPBS using Precellys ceramic beads (Precellys Lysing Kit, Cat# KT03961-1-001-2) for 2 × 30 sec at 6600 rpm on a Precellys homogenizer (Bertin Instruments, Cat# P000669-PR240A). For other experimental comparisons, spheroids were lysed using a TissueLyser II (Qiagen) for 50 sec (undifferentiated spheroids) or 1.5 min (differentiated spheroids) at 20 Hz. Lysates were centrifuged for 8 min at 5000 × g at 4°C. Pellets were resuspended in lysis buffer (10 mM Tris (Cat# 4855.2, Carl Roth), 1 mM EDTA, 0.3 M sodium acetate (Cat# 6779.1, Carl Roth), 1% SDS (Cat# 4360.2, Carl Roth) supplemented with proteinase K (0.2 mg/mL; Cat# 740506, Macherey-Nagel) and incubated overnight at 37°C.

##### DNA extraction

Following proteinase K digestion, RNase A (0.2 mg/mL; Cat# EN0531, Thermo Fisher Scientific) was added and samples were incubated at 37°C for 30 min. Tris-buffered phenol were added to the pellet, followed by gentle mixing and centrifugation for 5 min at 4000 rpm at RT. The aqueous phase was transferred to a new tube, mixed with chloroform–isoamyl alcohol (24:1) (Cat# 39554.02, Serva), and centrifuged for 5 min at 4000 rpm at RT. The DNA-containing upper phase was precipitated using 2.5 volumes of 100% ethanol and 0.1 volume of 3 M sodium acetate (pH 5.2). Samples were centrifuged for 30 min at 13,000 × g at 4°C. Pellets were washed with 70% ethanol, centrifuged for 4 min at 10,000 × g at RT, air-dried for 30 min, and dissolved in 50 μL TE buffer (1 M Tris, 0.5 M EDTA, pH 8.0) for 15 min at 37°C in a shaker at 500 rpm at 37°C.

##### RT-qPCR and mtDNA analysis

Quantification of mitochondrial and genomic DNA was performed using SYBR Green Master Mix (Cat# K0252, Thermo Fisher Scientific) with 2 ng of template DNA per 384-well. RT-qPCR was run on the LightCycler® 480 System (Roche Diagnostics) with LightCycler 480 SW 1.5 software for all LM and rosiglitazone experiments, or on the CFX384 Touch Real-Time PCR Detection System (Bio-Rad) with CFX Maestro software for all other experiments.

The mtDNA content was calculated as the mean of three mitochondrial gene targets (*CYTB*, *DLOOP* and *16S*) relative to the geometric mean of three genomic targets (*HBB*, *APP* and *B2M*). Data was analyzed using qBase+ software version 3.4 (Biogazelle). Primer sequences are provided in Key resources table.

#### Histology

##### Cryoembedding and freezing

Adipocyte spheroids were washed twice with 1× DPBS and transferred into plastic molds partially filled with pre-frozen Tissue-Tek O.C.T. compound (OCT; Cat# 45830, Histolab, Sweden). This partial fill prevented spheroids from settling at the edges. The spheroids were then fully embedded in OCT to position them centrally in the mold. Molds were frozen at – 100°C for 3 min using a Minitube Freezer (Minitube GmbH) and stored at –80°C until sectioning.

##### Cryosectioning

Before sectioning, molds were equilibrated overnight at –25°C to stabilize cutting conditions. The 10 μm thick cryosections were prepared using a Leica CM3050 cryostat (Leica Biosystems) at –30°C. Sections were mounted on microscope slides (Superfrost™ Plus, Thermo Fisher) and stored at –20°C until staining.

##### Hematoxylin and Eosin (H&E) staining

Cryosections were thawed at 4°C for at least 10 min and fixed with acetone (Cat# 9372.4, Carl Roth) at RT for 10 min. After air drying, differentiated adipocyte spheroids were stained with hematoxylin (Cat# 01820, Histolab) for 2 min. Sections were rinsed under tap water for 5 min, followed by a brief rinse in distilled water. Eosin staining (Cat# 1650, Histolab) was applied for 2 min. Sections were dehydrated through graded ethanol: 3×10 sec in 96%, 1×10 sec in 100%, 1×1 min in 100%, and 2×2 min in 100%. This was followed by 2×2 min in xylene (Cat# 9713.1, Carl Roth). Slides were mounted using ROTI®-Histokitt (Cat# 6638.2, Carl Roth GmbH), covered with a cover slip and stored at RT.

##### Imaging

Stained sections were imaged using a PreciPoint M8 digital microscope (PreciPoint GmbH) at 20× magnification. Images were acquired and viewed using ViewPoint Light software (PreciPoint GmbH).

### Quantification and statistical analyses

All statistical analyses were conducted using GraphPad Prism version 10.1.2. Outliers were identified using the ROUT method (Q = 1%) and excluded prior to analysis. Normality of data distributions was assessed using the Shapiro–Wilk test. For two-group comparisons, unpaired two-tailed t-tests were used when data followed a normal distribution, while Mann–Whitney U-tests were applied for non-normal distributions. Comparisons among three groups were analyzed using one-way ANOVA for normally distributed data or the Kruskal–Wallis test for non-normal data distribution. ANOVA was followed by Fisher’s Least Significant Difference (LSD) test, and Kruskal–Wallis by Uncorrected Dunn’s post hoc test to assess differences of planned comparisons among the groups. Sample sizes (N) for each experiment are specified in the corresponding figure legends. Results were considered statistically significant at a threshold of *p* < 0.05, and data are presented as mean ± SEM unless otherwise indicated.

## Supplemental information

Document S1. Figures S1–S5 and Table S1

Table S2. Analysis sequence for image analysis with Harmony 4.9, related to Figures 1–6
