# Supplementary material for: In vitro model of human subcutaneous adipocyte spheroids for studying mitochondrial dysfunction and mitochondria activating compounds
Source: iScience. 2025 Dec 29;29(2):114480. doi: 10.1016/j.isci.2025.114480 (PMC12856283; doi:10.1016/j.isci.2025.114480)
Supplement: Document S1. Figures S1–S5 and Table S1 [file mmc1.pdf]

iScience, Volume *29*

Supplemental information

In vitro model of human subcutaneous adipocyte spheroids for studying mitochondrial dysfunction and mitochondria activating compounds

Anita Wagner, Juulia H. Lautaoja-Kivipelto, Kalle Pehkonen, Antti Hassinen, Minna Kuusela, Lisa Röttger, Elena Herbers, Anna Ioannidou, Sophia Mädler, Ina Rothenaigner, Soumya Srinivasan, Sini Laasonen, M. Tanvir Rahman, Pinja Elomaa, Saija Kortetjärvi, Anneli Olsson, Olavi Ukkola, Kamyar Hadian, Matthias Mann, Hilkka Peltoniemi, Kirsi H. Pietiläinen, Martin Klingenspor, Kirsi A. Virtanen, Carolina E. Hagberg, and Eija Pirinen

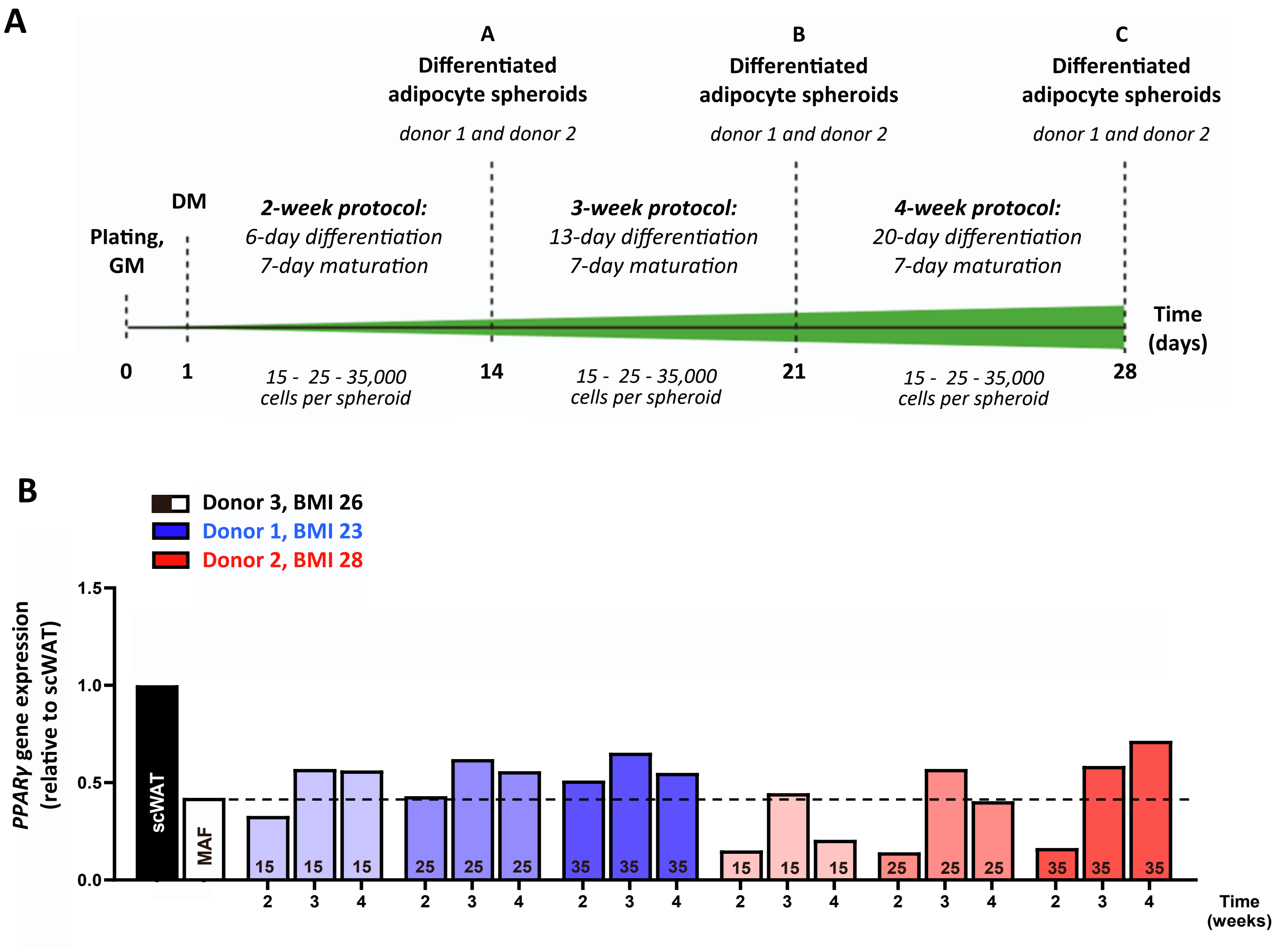

Supplementary Figure 1. Validation of the differentiation time and cell number for human subcutaneous white adipocyte spheroid model, Related to Figure 1. (A) Schematic presentation of the study design. (B) Gene expression of *PPARγ* in differentiated adipocyte spheroids of various sizes (15,000, 25,000 or 35,000 cells per spheroid) from the donors 1 and 2 after different differentiation time points (2, 3 or 4 weeks) and from scWAT and subcutaneous MAF from the donor 3, N = 1 per group. Data are presented as relative to scWAT = 1. Dashed line represents *PPARγ* expression level in MAFs. For the donor characteristics, see Table 1. Note that in contrast to our gold standard protocol, adipocyte spheroids analyzed in these experiments were not embedded into GFR-Matrigel.

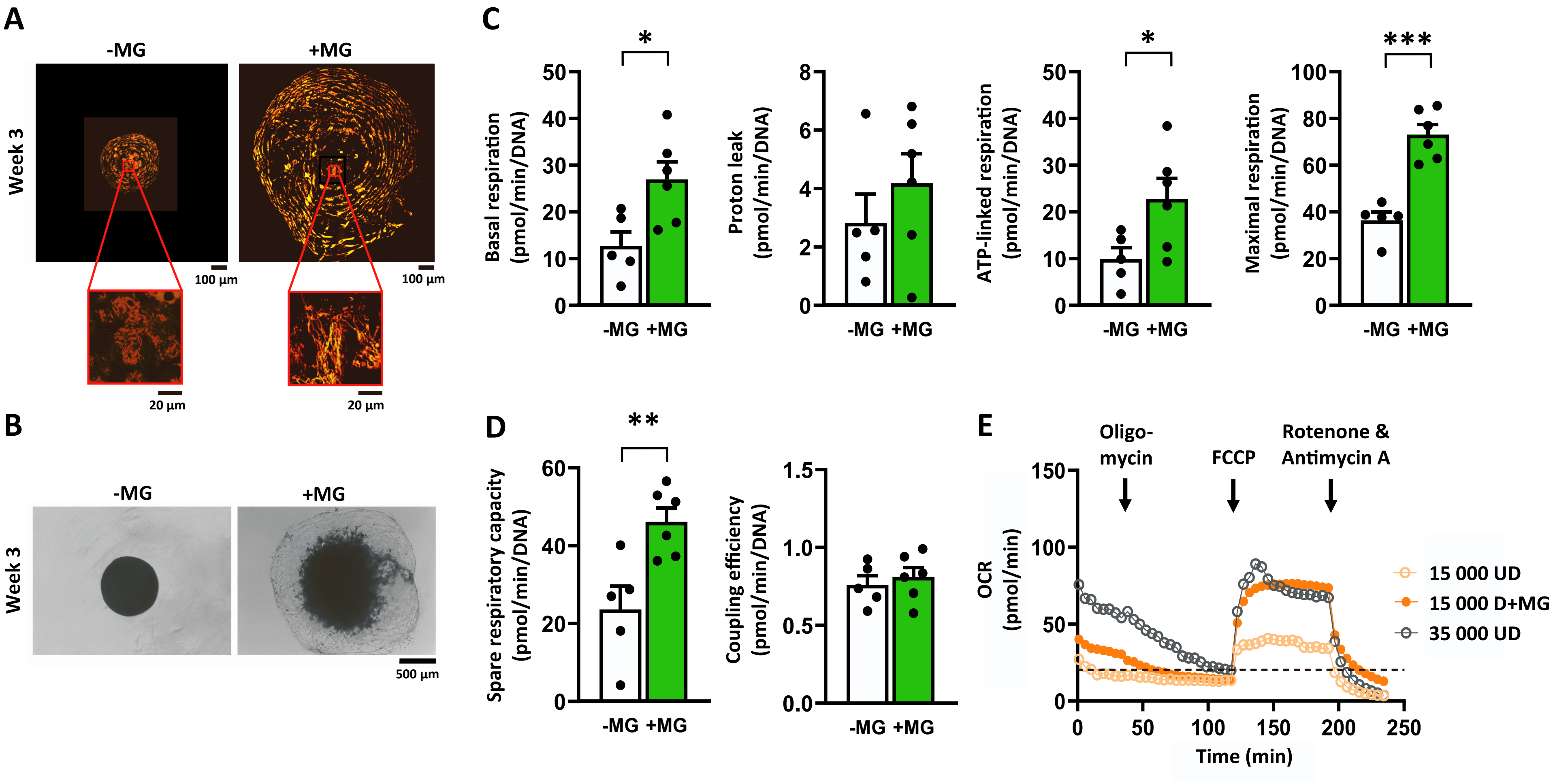

Supplementary Figure 2. Embedding of human subcutaneous white adipocyte spheroids into GFR-Matrigel improves mitochondrial network and respiration, Related to Figures 1 and 2. (A) Selected representative fluorescence live cell imaging of differentiated adipocyte spheroids derived from the donor 2 for the analysis of mitochondria (MitoTracker, yellow). On the left, adipocyte spheroid without (-MG) and on the right with (+MG) GFR-Matrigel embedding. Magnification 40x water immersion objective. Scale bar = 100 µm or 20 µm. (B) Selected representative bright-field images on the differentiated adipocyte spheroids derived from the donor 2 without and with GFR-Matrigel embedding. Scale bar = 500 µm. (C-D) Parameters of mitochondrial respiration, including basal respiration, proton leak, ATP-linked respiration, maximal respiration and spare respiratory capacity and coupling efficiency in differentiated adipocyte spheroids without or with

GFR-Matrigel embedding, N = 5-6 per group. (E) Raw oxygen consumption rate (OCR) values from undifferentiated (UD) and/or differentiated embedded in Matrigel (D+MG) (pre)adipocyte spheroids containing 15 000 or 35 000 cells per spheroid from the donor 2, N = 8-9 per group. For clarity, error bars are not shown. The dashed line represents the 20 pmol/min OCR level.

In (C-D), data are shown as means ± SEM with individual values. In (C-D), unpaired t-test was used. * $p < 0.05$, ** $p < 0.01$ and *** $p < 0.001$.

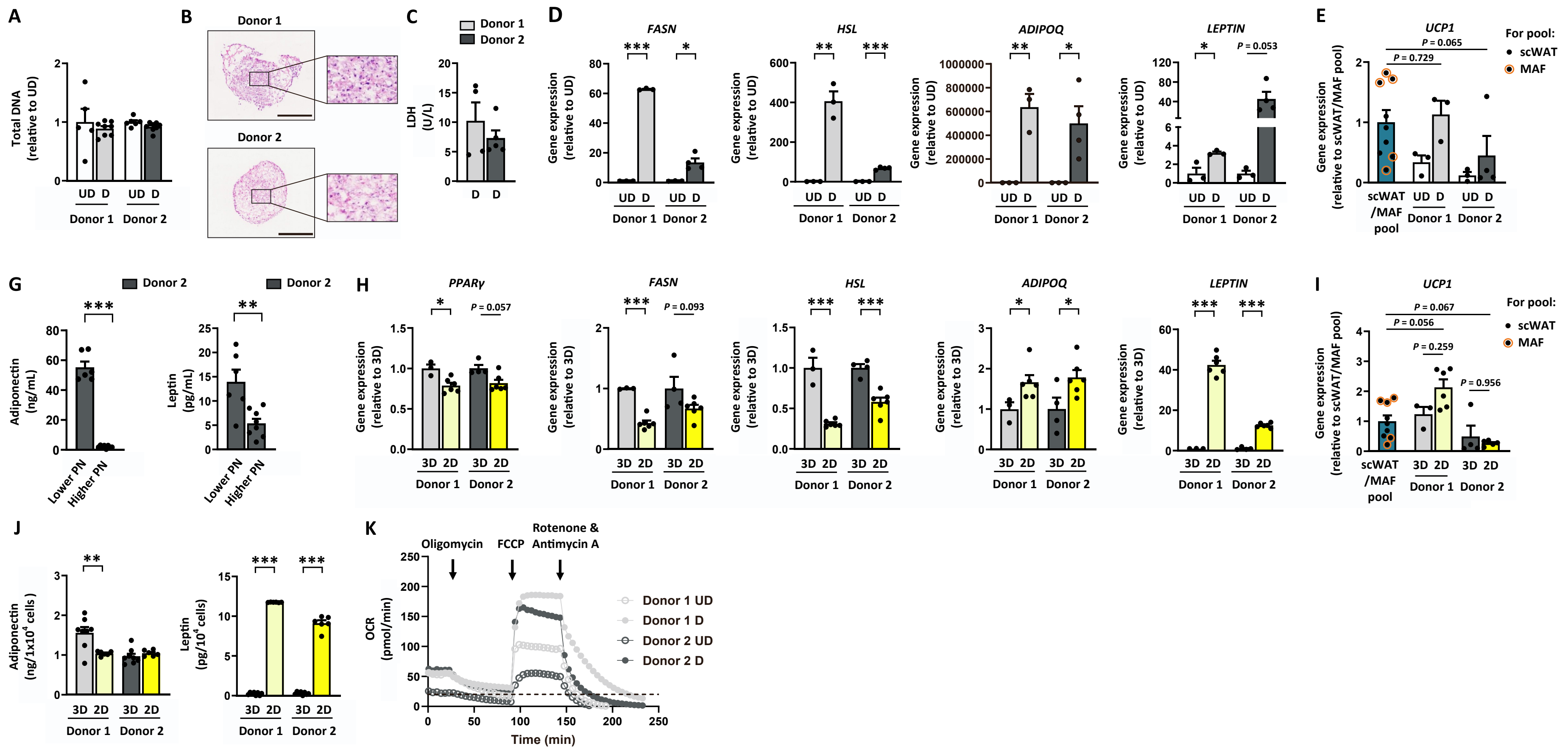

Supplementary Figure 3. Characteristics of undifferentiated (UD) and differentiated (D) (pre)adipocyte spheroids, Related to Figures 1 and 2. (A) Total DNA amount in UD and D (pre)adipocyte spheroids derived from the donors 1 and 2, N = 5-8 per group. (B) Selected representative cryosections with haemotoxylin and eosin staining images of the differentiated adipocyte spheroids from the donors 1 and 2. Scale bar = 500 µm. (C) Lactate dehydrogenase (LDH) enzyme activity measured from the media of differentiated adipocyte spheroids derived from the donors 1 and 2 after the 3-week differentiation period, N = 4-5 per group. (D) Gene expression of *FASN*, *HSL*, *ADIPOQ* and *LEPTIN* in UD and D (pre)adipocyte spheroids derived from the donors 1 and 2, N = 3-4 per group. Data are presented as relative to UD = 1. (E) Gene expression of *UCP1* in UD and D (pre)adipocyte spheroids relative to a pool of subcutaneous human white adipocyte tissue (scWAT) and subcutaneous human mature adipocyte fraction (MAF) samples, N = 3-9 per group. Note that scWAT/MAF pool consists of samples derived from the donors 4-

9 (Table 1). Data are presented as relative to scWAT/MAF pool = 1. (F) Media leptin concentration from UD and D (pre)adipocyte spheroids, N = 8 per group. (G) Media adiponectin and leptin concentrations from differentiated adipocyte spheroids derived from the donor 2 with lower (two to three) and higher (six) passage number (PN), N = 6-8 per group. (H) Gene expression of *PPARγ*, *FASN*, *HSL*, *ADIPOQ* and *LEPTIN* in three- and two-dimensional (3D and 2D, respectively) differentiated adipocytes (*i.e.* adipocyte spheroids and monolayer cultures, respectively) derived from the donors 1 and 2, N = 3-6 per group. (I) Gene expression of *UCP1* in 3D and 2D differentiated adipocytes derived from the donors 1 and 2 relative to a scWAT/MAF pool, N = 3-6 per group. Data are presented as relative to scWAT/MAF pool = 1. (J) Media adiponectin and leptin concentrations from 3D and 2D differentiated adipocytes derived from the donors 1 and 2 normalized to media volume and cell number, N = 6-8 per group. (K) Raw oxygen consumption rate (OCR) values from UD and D (pre)adipocyte spheroids from the donors 1 and 2, N = 13-32 per group. For clarity, error bars are not shown. For the normalized mitochondrial respiration data, see Fig. 2A-B. The dashed line represents the 20 pmol/min OCR level.

In (A) and (C-J), data are shown as means ± SEM with individual values. In (A), (C-D), (F-H) and (J), unpaired t-test or Mann-Whitney U-test was used. In (E) and (I), Kruskal–Wallis test followed by Uncorrected Dunn's test was used. * $p < 0.05$, ** $p < 0.01$ and *** $p < 0.001$.

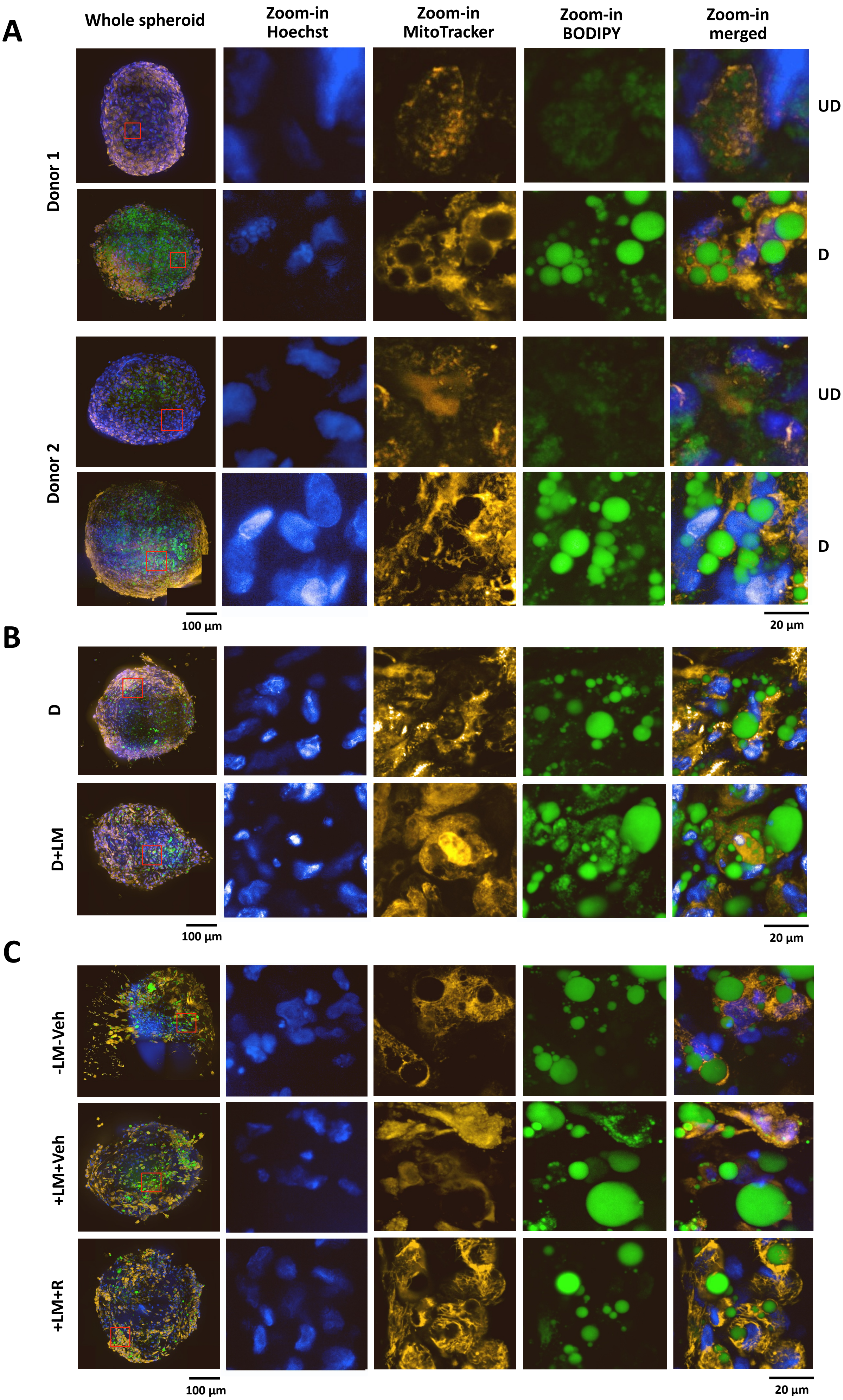
A
Whole spheroid
Zoom-in Hoechst
Zoom-in MitoTracker
Zoom-in BODIPY
Zoom-in merged
Donor 1
UD
D
Donor 2
UD
D
100 µm
20 µm
B
D
D+LM
100 µm
20 µm
C
-LM-Veh
+LM+Veh
+LM+R
100 µm
20 µm

Supplementary Figure 4. Morphological analyses of human (pre)adipocyte spheroids, Related to Figures 1-6. Selected representative fluorescence live cell imaging of the nuclei (Hoechst, blue), mitochondria (MitoTracker, yellow) and lipid droplets (BODIPY, green) and all three merged (whole adipocyte spheroid and zoom-in) in (A) undifferentiated (UD) and differentiated (D) (pre)adipocyte spheroids derived from the donors 1 and 2, (B) differentiated adipocyte spheroids administered without (D) and with lipid mixture (D+LM) derived from the donor 2 and (C) Differentiated adipocyte spheroids derived from the donor 2 administered without LM but with vehicle (-LM+Veh), with LM and vehicle (+LM+Veh) or with LM and 1 µM rosiglitazone (+LM+R) for 72h post differentiation. In (C), DMSO was used as vehicle. In the figures, the red rectangle represents the area from which the merged zoom-in has been taken. In whole spheroid images, scale bar is 100 µm and in merged zoom-ins, scale bar is 20 µm. Magnification is with 40x air objective.

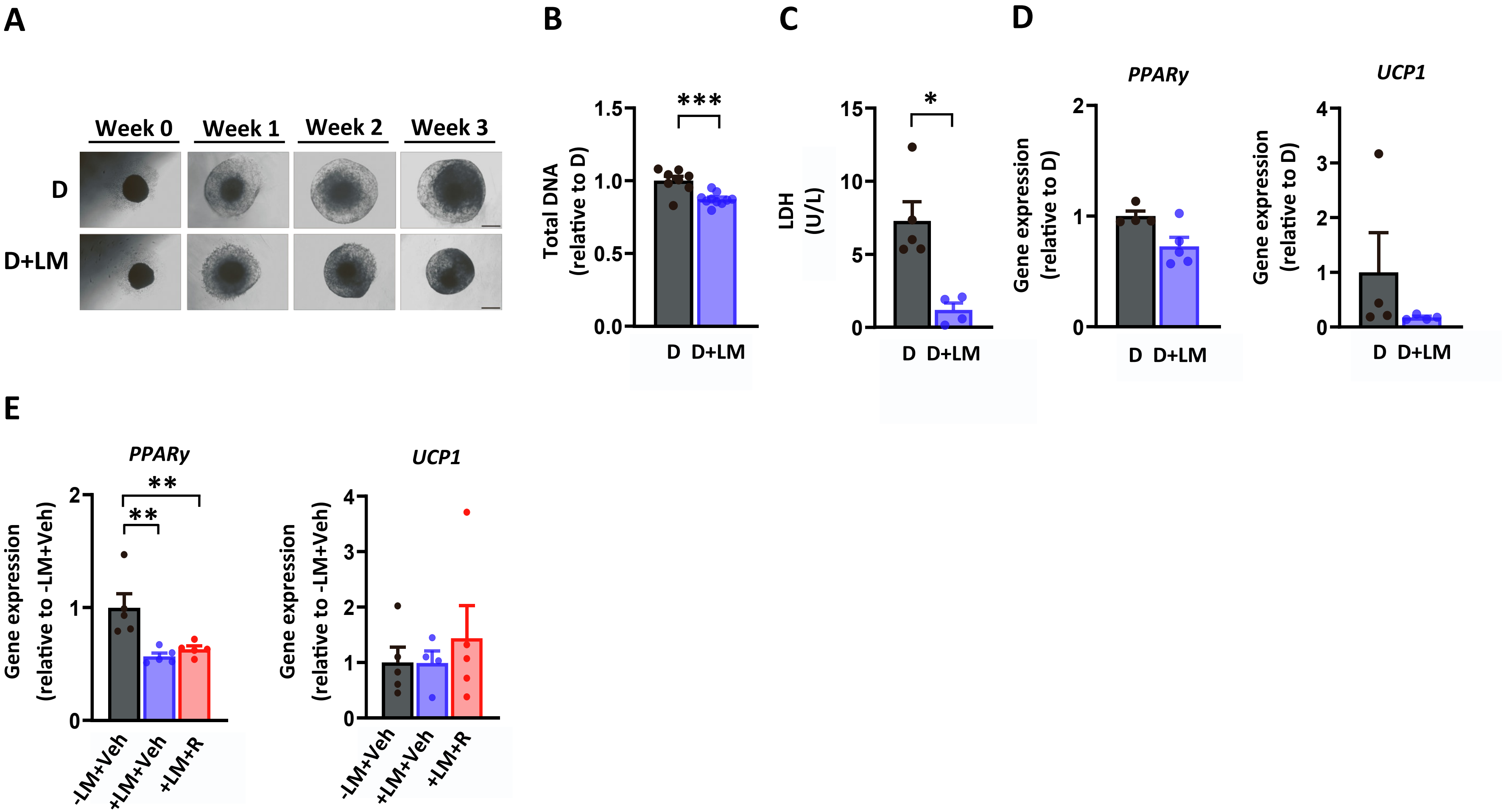

Supplementary Figure 5. The effect of lipid mixture and/or rosiglitazone treatment on DNA content, cell viability and gene expression, Related to Figures 3 and 5. Only differentiated adipocyte spheroids derived from the donor 2 were used in these experiments. (A) Selected bright-field images of the adipocyte spheroids without (D) and with lipid mixture (LM) administration (D+LM) during the 3-week differentiation period. Scale bar = 500 µm. (B) Total DNA amount in D and D+LM adipocyte spheroids, N = 8-10 per group. (C) Lactate dehydrogenase (LDH) enzyme activity measured from D and D+LM adipocyte spheroid media after the 3-week differentiation period, N = 4-5 per group. (D) Gene expression of *PPARγ* and *UCP1* in D and D+LM adipocyte spheroids, N = 3-4 per

group. (E) Gene expression of *PPARγ* and *UCP1* in differentiated adipocyte spheroids administered with vehicle (-LM+Veh), with LM and vehicle (+LM+Veh) or with LM and 1 μM rosiglitazone (+LM+R), N = 4-5 per group. In (B) and (D), data are presented as D = 1 and in (E), -LM+Veh = 1.

In (B-E), data are shown as means ± SEM with individual values. In (B-D), unpaired t-test or Mann-Whitney U-test was used. In (E), one-way ANOVA followed by Uncorrected Fisher's LSD was used. * $p < 0.05$, ** $p < 0.01$ and *** $p < 0.001$.

Table S1. Overview of selected ultra-low attachment (ULA) plate-based 3D culture models of human white subcutaneous adipocytes.

Cell type (source)	Plate type	Cell number	Scaffold	Vascularization	Total culture duration	Serum use in 2D and 3D cultures	Differentiation medium	Maintenance medium	Mitochondrial parameters analyzed	Reference
**Scaffold-free undifferentiated adipocyte spheroids**										
ASC (freshly isolated)	ULA 24-well plate	100,000/ well (many satellite spheroids/ well)	No	No	3 days	2D and 3D: 5% human PLT	Growth medium only[illegible] no differentiation or maintenance medium u[illegible]sed: αMEM medium, 1% P/S, 1% amphotericin B, 5 % human PLT, 20 FGF, 20 ng/mL EG[illegible]g/mL F, 2 µg/mL heparin		Yes; mitochondrial respiration, mtDNA copy number, mitochondrial content and morphology, OXPHOS complex protein expression levels	(1)
ASC (Promo Cell)	ULA 96-well plate	3,000/ spheroid	No	No	3 days	2D and 3D: 10% FBS	Growth medium only[illegible] no differentiation or sed: maintenance medium u25% mesenchymal stem cell growth mediu[illegible]n and 75% DMEM, 10% FBS, 1% P/S		Yes; mitochondrial respiration, mitochondrial content and morphology (TEM, confocal), OXPHOS complex protein and gene expression levels, drug responsiveness	(2)
**Scaffold-free differentiated adipocyte spheroids**										
ASC (freshly isolated) & NPAD cell line	ULA 96-well plate (preceeding hanging drop)	5,000-20,000/ spheroid	No	No	49 days	2D and 3D: 10% FBS	Commercially available[illegible]edium PGM-2, including bullet ki[illegible] (Lonza)		No	(3)
SVFs (freshly isolated)	ULA 96-well plate	5,000/ spheroid	No	No	42 days	2D: 10% FBS<br>3D: serum-free	William´s E, 1% P/S, 33 µM biotin, 17 µM pantothenate, 1.72 µM insulin, 100 nM DEXA, 500 µM IBMX, 125 nM transferrin, 2 nM T3, 10 nM hydrocortisone, 38.7 nM sodium selenite, 10 µM rosiglitazone	Serum-free William´s E without differentiation additives	No	(4)

SVFs (ZenBio)	ULA 96-well plate	5,000 or 10,000/ spheroid	No	No	21 days	2D: 10% FBS 3D: serum-free	Commercially available BB medium B: DMEM/F12 containing unknown concentration of biotin, pantothenate, insulin, DEXA, IBMX, and PPARγ agonist (Bonds Biosystems)	Commercially available BB medium C: DMEM/F12, 1 % P/S and unknown concentration of biotin, pantothenate, insulin, and DEXA (Bonds Biosystems)	No	(5)
SGBS cells & ASCs (freshly isolated)	ULA 96-well plate	20,000/ spheroid	No	No	12 days	2D: 10% FBS 3D: serum-free	DMEM/F12 1% P/S; 3.3 μM biotin, 1.7 μM pantothenic acid, 20 nM insulin, 25 nM DEXA, 250 μM IBMX, 10 μg/mL transferrin		No	(6)
**Scaffold-based differentiated adipocyte spheroids**										
SVF (freshly isolated)	ULA 96-well plate	25,000/ spheroid	GFR-Matrigel	Yes	17 days	2D: 10% FCS 3D: 2% FCS	Commercially available EGM2 (Lonza) & medium composition of αMEM, 2% FCS, 1% P/S, 5 μg/mL insulin, 10 μg/mL apotransferin, 0.2% intralipid, 50 ng/mL BMP7		No	(7)
SVF (Lonza)	ULA 96-well plate	10,000/ spheroid	GFR-Matrigel	Yes	40 days	2D: 10% FBS 3D: serum-free	Commercially available EGM-2 (Lonza)	Commercially available PGM-2 (Lonza)	No	(8)

αMEM, Minimum Essential Medium Eagle; 2D/3D, two/three dimensional; ASC, adipose-derived stem/stromal cells; BMP7, bone morphogenetic protein 7; DEXA, dexamethasone; DMEM, Dulbecco's Modified Eagle's Medium; DMEM/F-12, Dulbecco's Modified Eagle Medium/Ham's F-12; EGF, epidermal growth factor; EGM2, endothelial cell growth medium-2; FBS, fetal bovine serum; FCS, fetal calf serum; FGF, fibroblast growth factor; GFR, growth factor reduced; IBMX, isobutyl methylxanthine; mtDNA, mitochondrial DNA; NPAD, non-diabetic human preadipocyte cell line; OXPHOS, oxidative phosphorylation, PPARγ, peroxisome proliferator-activated receptor g, PGM-2, preadipocyte growth medium; P/S, penicillin/streptomycin; PLT, human platelet lysate; SGBS, Simpson-Golabi-Behmel syndrome; SVF, stromal vascular fraction-derived cells; T3, triiodothyronine and TEM, transmission electron microscopy.

References

1. **Rybkowska P**, **Radoszkiewicz K**, **Kawalec M**, **Dymkowska D**, **Zabłocka B**, **Zabłocki K**, **Sarnowska A**. (2023). The Metabolic Changes between Monolayer (2D) and Three-Dimensional (3D) Culture Conditions in Human Mesenchymal Stem/Stromal Cells Derived from Adipose Tissue. *Cells,* 12: 178, doi: 10.3390/CELLS12010178.

2. **Chang MM**, **Chu DT**, **Lin SC**, **Lee JS**, **Vu TD**, **Vu HT**, **Ramasamy TS**, **Lin SP**, **Wu CC**. (2025). Enhanced mitochondrial function and delivery from adiposederived stem cell spheres via the EZH2-H3K27me3-PPARγ pathway for advanced therapy. *Stem Cell Research and Therapy* 16: 1–21, doi: 10.1186/S13287-025-04164-1/FIGURES/7.

3. **Klingelhutz AJ**, **Gourronc FA**, **Chaly A**, **Wadkins DA**, **Burand AJ**, **Markan KR**, **Idiga SO**, **Wu M**, **Potthoff MJ**, **Ankrum JA**. (2018). Scaffold-free generation of uniform adipose spheroids for metabolism research and drug discovery. *Sci Rep* 8: 1–12, doi: 10.1038/S41598-017-19024-Z;TECHMETA.

4. **Shen JX**, **Couchet M**, **Dufau J**, **de Castro Barbosa T**, **Ulbrich MH**, **Helmstädter M**, **Kemas AM**, **Zandi Shafagh R**, **Marques M**, **Hansen JB**, **Mejhert N**, **Langin D**, **Rydén M**, **Lauschke VM**. (2021). 3D Adipose Tissue Culture Links the Organotypic Microenvironment to Improved Adipogenesis. *Advanced Science* 8: 2100106, doi: 10.1002/advs.202100106.

5. **Dariolli R**, **Nir R**, **Mushlam T**, **Souza GR**, **Farmer SR**, **Batista ML**. (2025). Optimized scaffold-free human 3D adipose tissue organoid culture for obesity and disease modeling. *SLAS Discovery* 31, 100218, doi: 10.1016/j.slasd.2025.100218.

6. **Mandl M**, **Heuboeck E**, **Benedikt P**, **Huber F**, **Mamunchak O**, **Grossmann S**, **Kotnik M**, **Hamzic-Jahic E**, **Bhogal CS**, **Lipp AM**, **Raml E**, **Zwerschke W**, **Wabitsch M**, **Voelkl J**, **Zierer A**, **Bernhard D**. (2025). The Capability to Undergo ACSL4-Mediated Ferroptosis Is Acquired During Brown-like Adipogenesis and Affected by Hypoxia. *Cells* 14: 1247, doi: 10.3390/CELLS14161247/S1.

7. **Muller S**, **Ader I**, **Creff J**, **Leménager H**, **Achard P**, **Casteilla L**, **Sensebé L**, **Carrière A**, **Deschaseaux F**. (2019). Human adipose stromal-vascular fraction self-organizes to form vascularized adipose tissue in 3D cultures. *Sci Rep* 9: 7250, doi: 10.1038/s41598-019-43624-6.

8. **Ioannidou A**, **Alatar S**, **Schipper R**, **Baganha F**, **Åhlander M**, **Hornell A**, **Fisher RM**, **Hagberg CE**. (2022). Hypertrophied human adipocyte spheroids as in vitro model of weight gain and adipose tissue dysfunction. *J Physiol* 600: 869–883, doi: 10.1113/JP281445.
